# Nanoparticle protein corona: from structure and function to therapeutic targeting

**DOI:** 10.1039/d2lc00799a

**Published:** 2023-01-19

**Authors:** Ghazal Bashiri, Marshall S. Padilla, Kelsey L. Swingle, Sarah J. Shepherd, Michael J. Mitchell, Karin Wang

**Affiliations:** a Department of Bioengineering, Temple University Philadelphia PA 19122 USA karin.wang@temple.edu; b Department of Bioengineering, University of Pennsylvania Philadelphia PA 19104 USA; c Abramson Cancer Center, Perelman School of Medicine, University of Pennsylvania Philadelphia PA 19104 USA; d Institute for Immunology, Perelman School of Medicine, University of Pennsylvania Philadelphia PA 19104 USA; e Cardiovascular Institute, Perelman School of Medicine, University of Pennsylvania Philadelphia PA 19104 USA; f Institute for Regenerative Medicine, Perelman School of Medicine, University of Pennsylvania Philadelphia PA 19104 USA

## Abstract

Nanoparticle (NP)-based therapeutics have ushered in a new era in translational medicine. However, despite the clinical success of NP technology, it is not well-understood how NPs fundamentally change in biological environments. When introduced into physiological fluids, NPs are coated by proteins, forming a protein corona (PC). The PC has the potential to endow NPs with a new identity and alter their bioactivity, stability, and destination. Additionally, the conformation of proteins is sensitive to their physical and chemical surroundings. Therefore, biological factors and protein–NP-interactions can induce changes in the conformation and orientation of proteins *in vivo*. Since the function of a protein is closely connected to its folded structure, slight differences in the surrounding environment as well as the surface characteristics of the NP materials may cause proteins to lose or gain a function. As a result, this can alter the downstream functionality of the NPs. This review introduces the main biological factors affecting the conformation of proteins associated with the PC. Then, four types of NPs with extensive utility in biomedical applications are described in greater detail, focusing on the conformation and orientation of adsorbed proteins. This is followed by a discussion on the instances in which the conformation of adsorbed proteins can be leveraged for therapeutic purposes, such as controlling protein conformation in assembled matrices in tissue, as well as controlling the PC conformation for modulating immune responses. The review concludes with a perspective on the remaining challenges and unexplored areas at the interface of PC and NP research.

## Abstract 2

Representation of the influence of biological and nanoscale factors on protein corona conformation and function in a healthy *versus* a pathogenic biological environment.

## Graphical abstract

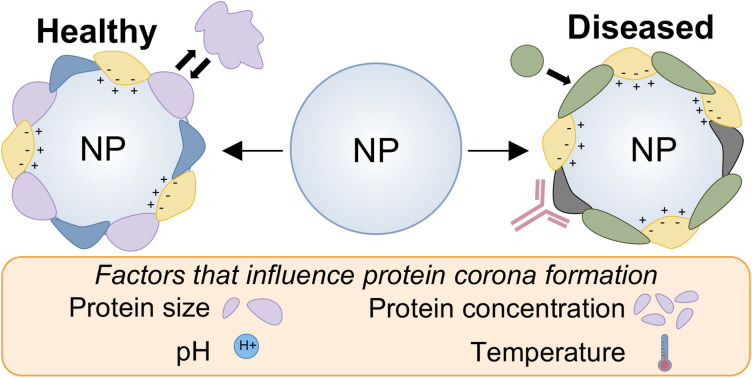

## 1. Introduction

Decades of intense drug discovery research have yielded a plethora of potent therapeutics for a variety of diseases. Unfortunately, most drugs fail to reach government approval due to poor efficacy and toxicity.^1,2^ A major cause of this failure is the incompatibility of many therapeutics with the anatomy and physiology of the human body.^3^ Hydrophobic drug candidates suffer from poor bioavailability while biological therapeutics such as proteins and nucleic acids are rapidly degraded. Since modulating the chemical structure of a drug can result in lower efficacy, researchers and clinicians have turned to nanoparticles (NPs), which are nano- or micro-sized vehicles that protect and deliver the desired therapeutic.^4^ NPs offer more favorable pharmacokinetics while still preserving the intrinsic structure of the drug.^5^ The first FDA-approved nanocarrier–drug combination is Doxil, a liposomal formulation of doxorubicin that received approval in 1995 for the treatment of a variety of cancers.^6^ More recent examples include the COVID-19 vaccines, Spikevax and Comirnaty, which are lipid nanoparticle formulations that encapsulate spike protein mRNA.^7^

The amalgamation of nanotechnology and drug discovery has produced several FDA-approved therapies; however, NPs have several obstacles that have prevented their broader utilization.^8^ This includes their own intrinsic toxicity, complexity, and the lack of one-size-fits-all NPs, the latter meaning that most therapeutics must have their own tailored drug delivery vehicle.^9^ Additionally, NPs can lower the bioavailability and efficacy of a drug, especially as the drug can become trapped in the particle. As with therapeutics, NP complications are often a result of biological barriers, and their successful translation to clinics heavily depends on controlling *in vivo* factors affecting biodistribution, blood residence, and targeting specific tissues and cells.^10^ One important, yet often overlooked factor that governs NP success is the role of the protein corona (PC). A PC is the layers of proteins that adsorb onto the NP after administration.^11,12^ This occurs due to the high concentration and wide number of freely diffused proteins in the body. Often, the PC is subdivided into two segments, hard and soft coronas.^13^ The hard corona is the inner-most layer and has proteins that bind more tightly. The soft corona is composed of loosely bound proteins that attach to the hard corona. However, contrary to studies reporting the formation of a multilayer PC,^14–16^ other studies have demonstrated the formation of a monolayer PC.^17^ It has been recently hypothesized that the soft and hard coronas can be made of the same proteins with different binding strengths, and that the soft corona refers to proteins capable of both transient and stable interactions.^18^ Therefore, hard and soft coronas can coexist in a monolayer that becomes less dense as soft corona proteins dissociate and partially expose bare NP sites, favoring non-specific NP–cell interactions dependent on the surface chemistry of the NPs. It should be noted that this is an intriguing area of PCs that is still being investigated.

The composition of a PC strongly depends on the shape, size, and molecular composition of the NP.^19^ For instance, large hydrophobic particles will form distinct PCs compared to small positively charged NPs.^20^ The PC can drastically affect the stability and biodistribution of the drug delivery vehicle.^19,21^ For example, NPs that bind to apolipoprotein E (APOE) are often trafficked to the liver, which can be exploited for therapeutics designed for hepatic diseases.^22^ However, for drugs that need to be delivered elsewhere, APOE binding can lead to liver toxicity. The precise role of each protein in the PC and mechanisms involved in determining NP fate are still being investigated. While most research on PCs has focused on identifying the proteins adsorbed onto NPs, recent studies have demonstrated that understanding the conformation of proteins on the PC, not solely their identity, is essential in understanding how PCs impact NPs.^23^ For instance, NPs with similar PCs can be trafficked to different locations, as the same protein may be folded in unique manners and thus present distinctive amino acids on the surface of the PC that are recognized by different receptor and carrier proteins.^24^ PC conformation also impacts the aggregation behavior and overall toxicity of the NPs.^25^

Conformational changes can arise from interactions with the NP, but also with interactions between neighboring proteins on the PC.^26^ This creates a dynamic system as proteins continuously adsorb and desorb from a PC.^27^ Additionally, the exact folding of a specific protein on a PC is more nuanced than a simple binary of folded or unfolded, as the tertiary structure of a protein may be altered in subtle ways, such as forming β-sheets instead of α-helices. Due to the complexity of the PC, this review aims to provide an analysis of modern studies on the impact of protein conformation on NPs, specifically as it relates to the influence of the biological environment and NP characteristics on protein conformation and corona formation. The review will also include instances in which protein conformation is leveraged to enhance NP efficiency, as well as a perspective on the future of this burgeoning area for therapeutic purposes.

## 2. Biological factors that influence protein corona conformation

### a. A nanoparticle's journey *in vivo*

**Fig. 1:**
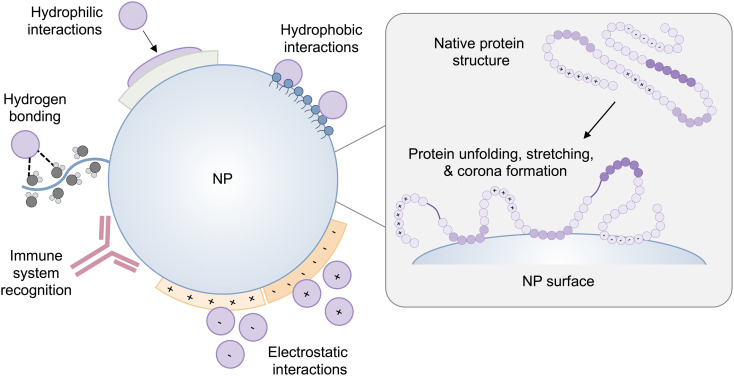
A schematic representation of protein–NP interactions and potential changes in protein conformation on the surface of the NP. The NP-induced protein conformational changes can cause proteins to expose cryptic binding sites, affecting protein function as well as NP fate *in vivo*.

The conformation of a protein in its free energy minimum in solution does not always correlate to its free energy minimum when it comes into contact with a surface.^28^ Accordingly, proteins undergo conformational changes on the surface of NPs as well as solid surfaces. The secondary structure of proteins, including α-helices and β-sheets, is stabilized with hydrogen bonds and combined hydrophobic interactions and hydrogen bonds, respectively.^29,30^ Many proteins form a tertiary structure in which the hydrophobic interactions are buried in a hydrophobic core, which is encapsulated by electrostatic interactions and hydrogen bonds between side-chain amino acid residues. Furthermore, van der Waals interactions serve to maintain the folded configuration of a protein. Therefore, when proteins approach a surface, adsorption forces governing protein–surface interactions can easily disrupt these non-covalent interactions in the protein structure, resulting in conformational changes or the collapse of protein structure – unfolding^31,32^ (Fig. 1). Due to their folded configuration, proteins include the distribution of hydrophilic, hydrophobic, positively charged, and negatively charged side chains.^28^ As a result, when a protein comes into contact with a hydrophilic surface, it undergoes conformational and orientational changes to expose its hydrophilic patches.^28,29^ On a hydrophobic substrate, proteins expose their hidden hydrophobic regions in their structure, and in the case of charged surfaces, proteins tend to reveal regions that have opposite charges to the surface.

NP–protein interactions differ depending on the type of NP, composition, and distinct physiochemical properties such as size, curvature, shape, and surface charge.^33,34^ The intrinsic stability of a protein determined by its secondary structure also impacts its interaction with NPs and the extent to which a protein undergoes conformational changes on the surface of NPs.^28,29,35^ Interestingly, NP-induced conformational changes in proteins are a double-edged sword. On the one hand, conformational changes of adsorbed proteins may impair protein functionality, which can have repercussions on the interaction of NPs with cells, or they may cause proteins to expose hidden binding sites triggering an immune response^31^ (Fig. 1). On the other hand, surface-induced conformational changes of proteins can be leveraged to induce the desired cell signaling for therapeutic applications, which is discussed in section 4.

**Fig. 2:**
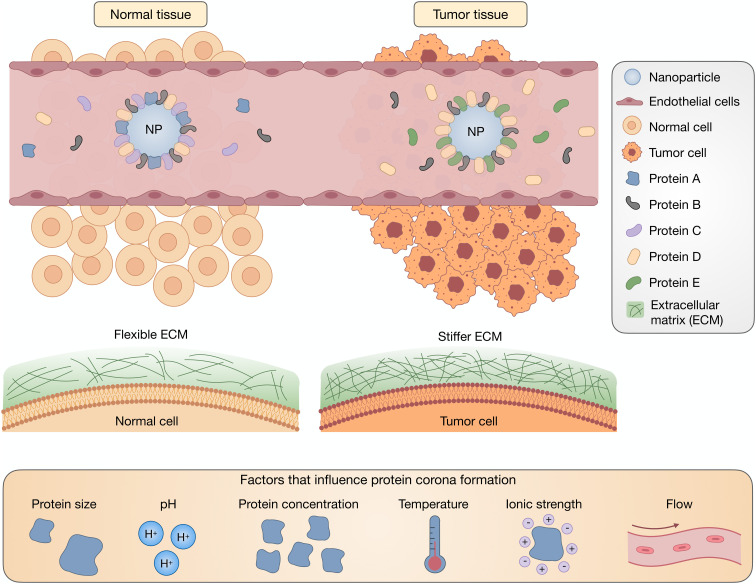
Biological factors affect the evolution of the PC, especially the conformation and orientation of PC-associated proteins. During the journey of NPs *in vivo*, depending on their route of administration and the overall condition of the body (normal/pathogenic), NPs interact with different types of proteins of different sizes and concentrations. Variations in environmental parameters such as pH, ionic strength, temperature, and shear flow also affect PC structure as NPs travel *in vivo*.

Traditionally, the biodistribution of a NP has been assumed to be due to its architecture and size; however, a growing body of literature has supported the idea that the PC strongly influences the locations in which NPs accumulate.^36,37^ Plasma proteins that adsorb onto NPs can result in their delivery to specific organs, the most common being the liver and spleen. This can be a benefit for therapies that require hepatic or splenic delivery but also can be an obstacle when other organs are the target. The composition of a PC depends on the route of administration, which reinforces the significance of exposure order in the development of a PC.^38^ Intravascularly injected NPs are first exposed to blood plasma proteins, such as albumins, fibrinogen (FBG), plasma fibronectin (FN), and globulins, with the most prevalent being serum albumin, before encountering extracellular matrix (ECM) proteins such as collagen and FN (Fig. 2). Extravascularly injected NPs pass through the skin and muscles before being absorbed. For tumors, NPs interface with the stiff ECM around the tumor tissue before reaching the cellular target site. Although much research has reported on the formation of blood plasma PCs, the formation of ECM and cellular protein coronas, and the order of exposure for NPs have yet to be investigated. In Table 1, we have listed some proteins frequently discussed in PC-related studies.

**Table 1:** Proteins commonly used in PC-related studies

Protein type	Molecular weight (kDa)	Isoelectric point (pI)	Location	Human plasma concentration	Main functions	Ref.
Human serum albumin	∼67	4.7	Blood stream	35–50 mg mL^−1^	Transport-related proteins	39
					Maintaining osmotic blood pressure	
Bovine serum albumin	∼69.3	4.7	Blood stream of cow	N/A	Regulation of the colloidal osmotic pressure of blood	40
			Whey component of bovine milk			
Fibronectin	∼500	5.6–6.1	Blood stream	300 to 400 μg mL^−1^	Cell adhesion, spreading, proliferation, migration, apoptosis, wound healing, and disease progression	41–43
			ECM			
Fibrinogen	∼340	5.5–5.8	Blood stream	1.5–4.0 g L^−1^	Blood clot formation	44, 45
					Wound healing	
					Inflammation	
					Blood vessel growth	
Factor XII	∼80	6.1–6.5	Bloodstream (it circulates as a zymogen, an inactivated enzyme)	15–47 μg ml^−1^	An enzyme circulating in the form of zymogen in blood and capable of initiating the clotting and fibrinolytic upon activation	46
von Willebrand factor	∼500–20 000	5.7–5.9	Blood stream	5–10 mg l^−1^	Hemostasis	47
					Platelet adhesion and aggregation during wound healing	
Immunoglobulin G	∼150	5.9–6.1	Blood stream, extracellular fluid	8–17 mg mL^−1^	Humoral immunity	39
Transferrin	∼80	∼6	Blood stream	200–400 mg dL^−1^	Transporting iron	48
Plasminogen	92	5.6	Blood stream	200 mg L^−1^	Breaks down fibrin blood clots	49
Hemoglobin	∼65	6.3	Red blood cells in blood stream	N/A	Transporting oxygen and carbon dioxide	50, 51
Lysozyme	∼14	11.1	Tears of the lacrimal glands of animals nasal mucus	N/A	Antimicrobial activity	52, 53
			Gastric secretions and egg white		Modulating the host immune response to infection (innate immunity)	
β-Lactoglobulin	18.4	5.2	Bovine milk and whey	N/A	Transporting hydrophobic molecules	52
Myoglobin	∼17	6.8–7.4	Heart and skeletal muscles cells, blood stream (only in case of muscle damage)	6–85 × 10^−9^ g mL^−1^	Transporting oxygen	54, 55
Apolipoprotein E	∼34	∼5.3	Blood stream	5 mg/100 ml	A key regulator of plasma lipid levels	56, 57
			Interstitial fluid and lymph		Homeostatic control of plasma and tissue lipid content	
Apolipoprotein A1 (ApoA1)	28.3	5.0–5.5	Blood stream	0.8–1.7 mg mL^−1^	Lipid metabolism	39
					Transport-related protein	
Collagen type I	300	∼7.2	ECM	N/A	Supporting mechanical strength of tissues and organs	58, 59
Apolipoprotein J (clusterin)	∼70–80	4.9–5.4 (for each subunit)	Blood stream, urine, breast milk, semen, and cerebrospinal	2–70 mg L^−1^	Lipid transportation	60, 61
					Tumor growth	
					Cell adhesion	
					Tissue remodeling	
					Immune system regulation	
					Oxidative stress	
					Amyloid associated protein	
Insulin	∼5.8	5.4	Blood stream	∼0.34 mU ml^−1^	Controlling the blood content of glucose	62
γ-Globulins	155–160	6.8–6.9	Blood stream	25 mg mL^−1^	Defense mechanism	63
					Transportation	
Prothrombin	∼70	∼5.2	Blood stream	1 × 10^−4^ g mL^−1^	An inactive precursor to thrombin, an essential component of the blood-clotting	64, 65

PC formation is strongly impacted by environmental conditions such as pH, ionic strength, temperature and shear flow, as well as protein concentration, size, and glycosylation (Fig. 2). Not only does the identity of the proteins change, but protein conformation and orientation are also affected.

### b. Effect of pH and ionic strength

The administration route for nanomedicines differs depending on the type of disease for which they are intended. As a result, nanomedicines can enter and pass through a variety of biological environments. One important physiological parameter is pH, which varies depending on the location within the body, and even the location within the cell.^66^ Additionally, the pH of the environment fluctuates depending on the state of the disease. For instance, the extracellular environment of malignant tumors is acidic (pH 6.5–6.9), while tissues under normal physiological conditions are neutral (pH 7.2–7.4).^67^ The local environment in bacterial infections is also acidic;^68,69^ however, the pH of a wound surface rises following an injury compared to intact skin with its acidic pH.^70^ The wound environment can become alkaline or acidic during the healing process, depending on the pathophysiology of the wound. Alkaline wounds have been attributed to non-healing wounds, while acidic wounds have been associated with healing wounds.

A protein's binding and structure change in response to pH, which can dramatically alter its stability and biological activity.^71–75^ Protein adsorption is preferred at its isoelectric point (pI) because electrostatic repulsion and protein–protein interactions are minimized, allowing compact protein molecules to tightly pack onto the surface of the NP.^40,76–79^ Along with pH, ionic strength is an essential parameter that greatly influences the architecture and biological activity of the PC. Ionic strength varies depending on the location, for example, blood is 150 mM, while bile is 3–15 mM.^80–83^ Therefore, understanding the role of pH and ionic strength in PC formation and conformation is critical and should be considered while designing nanomedicines.

FN is a protein found in plasma and the ECM and has binding sites for cell receptors, growth factors, and other matrix proteins. FN plays an essential role in embryogenesis, tissue regeneration, and disease progression.^41^ Changes in the molecular conformation of FN can expose buried molecular recognition sites or even disrupt binding sites, thereby changing its binding interactions and physiological functions.^84–86^ Plasma FN has a compact yet flexible structure under physiological conditions, whereas raising the pH or ionic strength causes it to unfold.^87^ Interestingly, lowering the pH to 2.8 or lowering the ionic strength also unfolds FN.^88^

The conformation of FN directly accessing NP surfaces was evaluated against that of FN attaching to protein corona-coated NPs by using Förster resonance energy transfer (FRET)-labeled FN on citrate-coated and pre-corona-coated gold nanoparticles (AuNPs) under acidic (pH 6.5) and physiological conditions (pH 7.4). It was observed that in acidic pH, less FN unfolding was detected on the surface of citrate-coated AuNPs than at physiological pH, which could be attributed to the lower affinity of FRET-FN for AuNPs under acidic conditions.^86^ However, this may be partially attributed to pH-sensitive changes in fluorescence emission, which could account for some of the observed alterations in the FRET ratio. Additionally, the disparity between the level of FRET-FN unfolding under acidic and physiological conditions is reduced with increasing AuNP concentration. Similarly, there is reduced FRET-FN adsorption and unfolding on pre-corona-coated AuNPs at acidic pH than under physiological conditions. Citrate-coated AuNPs, where FN can directly access the surface of NPs, induce a higher level of unfolding in FRET-FN than corona-coated NPs, where FN molecules interact with pre-adsorbed FN molecules. It suggests that the greater AuNP surface availability promotes FN unfolding. Additionally, the findings reveal that in the acidic tumor microenvironment, FN in the PC is likely to undergo less unfolding, which could have implications for controlling NP–cell interactions. Future research should investigate the effect of pH on the conformational states of FRET-FN in various biological environments and the accessibility of specific binding sites for NP surfaces.

Due to the widespread use of human serum in biomedical applications, determining the effect of pH on HSA and BSA structural changes on the surface of NPs is critical.^89^ Although BSA is not found in the human body, it is widely used in basic research studies to understand the fundamentals of the PC rather than translating it into clinical studies. The reason for this is that, when compared to HSA, BSA is less expensive, easier to obtain, and has similar physiochemical properties. Recently, the stability of HSA-coated magnetic iron oxide nanoparticles (MNPs) was evaluated in phosphate buffer at pH 6.0, 6.6, and 7.5, and ionic strengths of 0.15 and 0.30 M NaCl.^89^ At pH 6 and 0.05 M phosphate buffer, HSA coating on MNPs had the highest stability and the least exchangeability. However, increasing the ionic strength, as well as pH, decreased the stability of adsorbed HSA. Initial buffer conditions likely determine the conformational rearrangements of HSA molecules to expose distinct preferred binding sites for the surface of MNPs.

Along with changes in binding, there are also significant pH-dependent changes in the secondary and tertiary structures of BSA conjugated to AuNPs.^90^ BSA PCs adsorbed on TiO_2_ and SiO_2_ NPs have reduced coverage at lower pH, regardless of the type of NP.^91^ Furthermore, BSA adsorption on TiO_2_ NPs is significantly higher than that on SiO_2_ NPs, which could be due to the higher surface hydroxyl density of TiO_2_ NPs, which promotes hydrogen bonding with BSA, and diminishes as the pH drops. This emphasizes the importance of NP surface chemistry on PC formation, where seemingly small changes in elemental composition induce dramatic affects. Regardless of the pH and the type of NP, however, the secondary structure of BSA changes upon adsorption onto the NPs. Interestingly, adsorption of BSA onto TiO_2_ NPs results in complete denaturation at acidic pH, whereas adsorption onto SiO_2_ NPs induces an extended conformation. Therefore, the surface chemistry of NPs, as well as the pH of the solution, influences the structural alterations of proteins on the corona.

The impact of pH on BSA corona formation is also relevant to solid lipid nanoparticles (SLNs).^66^ At pH 6, electrostatic interactions govern BSA–SLN interactions, whereas van der Waals forces and hydrogen bonding interactions are dominant at pH 7.4. The interaction of BSA with SLNs results in secondary structural alterations of BSA. The BSA corona formation at pH 6 triggers the aggregation of SLNs and decreases their uptake into B16 murine melanoma cells. The aggregation is attributed to additional positive charges of BSA and its more compact structure at a lower pH, increasing its adsorption and weakening the electrostatic repulsion between SLNs. However, at pH 7.4, a stronger repulsion between BSA and SLNs reduces BSA adsorption, which results in more dispersed SLNs and a lower uptake in macrophages. The pH of the environment affects not only the surface charge but also the structure of proteins associated with the PC, which has downstream consequences for protein adsorption onto NPs and the overall fate of the NPs.

In the same vein, protein structural changes caused by pH fluctuations can impact the colloidal stability of NPs.^51^ At alkaline and neutral pH, hemoglobin (Hb) adsorption on AuNPs modified with three different capping ligands results in colloidally stable bioconjugates. Interestingly, the secondary structure of Hb remains unchanged after interacting with the AuNPs in a colloidally stable condition. Nevertheless, independent of the presence of capping ligands, Hb-AuNPs bioconjugates aggregate at pH 4, and the secondary structure of Hb on the surface of AuNPs slightly changes. Therefore, maintaining the structure of proteins in the PC is crucial for keeping NPs colloidally stable.

**Fig. 3:**
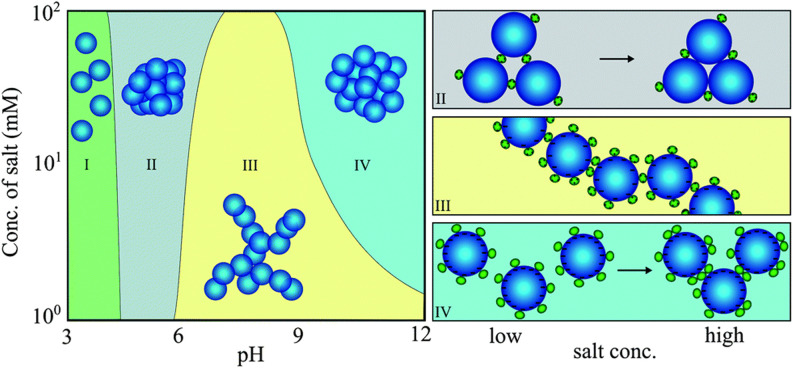
Protein-induced bridging aggregation of SiO_2_ NPs as a result of lysozyme adsorption at different pH values and salt concentrations. Reprinted with permission from ref. 93. Copyright 2014. Royal Society of Chemistry.

Another essential factor in the context of protein–NP interactions is the ionic strength of the solution. For example, although minor blood pH variations and increases in glucose concentration do not affect the binding of gamma-FBG peptides on SiO_2_ NPs, increasing the ionic strength weakens their binding by shielding electrostatic interactions.^92^ Protein-triggered aggregation of SiO_2_ NPs is dependent on both ionic strength and pH, as determined using lysozyme (Lyz) as a model (Fig. 3).^93^ Interestingly, pH, rather than ionic strength, is responsible for protein binding to SiO_2_ NPs. At low pH, where SiO_2_ NPs are almost uncharged, Lyz does not adsorb onto NPs (region I). At pH 5, the binding of a few Lyz proteins screens the repulsive interaction between weakly charged SiO_2_ NPs, resulting in NP aggregates with high packing density (region II). Under pH conditions greater than 6, the repulsive interactions between Lyz adsorbed on neighboring SiO_2_ NPs lead to loose NP aggregates (region III). However, as more salt is added, more repulsive interactions are shielded, leading to densely packed NP aggregates (region IV). This implies that protein-induced bridging aggregation of NPs is governed by pH and the ionic strength of the solution.

Comparable results in terms of the shielding impact of electrolytes are seen on lysozyme and β-lactoglobulin (β-LG) interactions with SiO_2_ NPs.^52^ Similarly, at low ionic strength, protein adsorption to the hydrophobic surface of sulfated (PS-OSO_3_H) NPs is irreversible, as there is no NP aggregation due to high NP–NP repulsion.^17^ However, charge screening in NP–protein and NP–NP interactions at high ionic strength leads to protein-triggered NP aggregation. A significant amount of protein-mediated NP agglomeration can occur with the adsorption of one transferrin (Tf) molecule on a single PS-OSO_3_H NP. Nevertheless, due to colloidal stabilization provided by the protein shell, NP surfaces are completely passivated against agglomeration if the number of bound proteins exceeds a certain threshold level.

Phosphate molecules are present in blood and buffer the pH of the environment.^40^ Since phosphate can co-adsorb on the surface of NPs, it can impact the adsorption and structure of proteins. The co-adsorption of phosphate with BSA on the surface of TiO_2_ NPs affects the affinity, adsorption, and conformation of BSA in a pH-dependent manner.^40^ At pH 7.4 and 4.5, phosphate has an insignificant effect on the secondary structure of adsorbed BSA. In contrast, at pH 2, the presence of phosphate significantly restricts BSA denaturation on the surface of the particles. This can be attributed to the blocking of the active sites on TiO_2_ NPs by phosphate molecules, preventing BSA from directly interacting and subsequently expanding its structure. Likewise, phosphate pre-adsorption on hematite (α-Fe_2_O_3_)NPs reduces protein surface coverage, slows the protein-specific kinetics of BSA and β-LG, and restricts secondary structural changes in proteins.^94^ Phosphate's ability to attenuate the α-Fe_2_O_3_NP-induced secondary structural changes of proteins is attributed to its induction of steric constraints or bridging and ternary complex formation, suggesting phosphate as a potential agent in attenuating adsorbed protein denaturation, particularly at low surface coverage.

Variations in pH also affect the evolution of the hard and soft PC layers.^16^ For instance, oxyhemoglobin (oxyHb) forms a hard PC on SiO_2_ NPs at pH 7, while it forms a soft PC of weakly bound proteins at pH 9 due to electrostatic repulsion between negatively charged oxyHb and the anionic siloxide groups at the surface of SiO_2_ NPs.^95^ In the absence of NaCl and at a pH less than the pI, electrostatic attraction between SiO_2_ NPs and myoglobin leads to a hard monolayer PC formation with randomly oriented proteins due to their symmetric charge distribution.^16^ However, the addition of NaCl screens the electrostatic repulsion between proteins adsorbed onto NPs and free proteins in bulk, resulting in the formation of an extra soft PC. This is lost when the pH becomes greater than the pI. At this pH, both the NPs and myoglobin are negatively charged, indicating that the formation of a hard PC monolayer is mediated by a charge regulation mechanism of the myoglobin amino acid residues, which leads to the adsorption of proteins onto NPs in a preferred orientation. Likewise, increasing the pH from acidic conditions to neutral and alkaline conditions increases the maximum amount of BSA and IgG proteins that can adsorb onto AuNPs, resulting in a transition from a monolayer PC to a multilayer PC structure.^15^ Interestingly, this transition is due to the changes in the properties of the NPs rather than the changes in the state of protein molecules in response to pH changes. At all pH and protein concentrations, IgG adsorbs onto AuNPs effectively. While BSA's effective binding occurs at all protein concentrations under acidic conditions, it only occurs at concentrations greater than 10 μg mL^−1^ under alkaline conditions.

The hard and soft PC formation can also be distinguished by altering the ionic strength of the medium.^83^ At low ionic strength, β2-microglobulin (β2m) interaction with citrate-stabilized AuNPs (Cit-AuNPs) leads to the formation of a tight layer of PC with a longer residence time. However, increasing the ionic strength results in a labile and soft β2m PC. This suggests that electrostatic forces govern Cit-AuNPs and β2m interactions. Thus, in the case of stable NPs and proteins, raising the ionic strength would avoid a hard PC formation and subsequent unfolding of proteins on the surface of NPs, although this requires further exploration.

Controlling the orientation of surface-immobilized proteins regulates their biological activity and is an important consideration when designing new biomaterials.^96^ For example, the antigen-binding capacity of IgG adsorbed onto AuNPs is pH-dependent.^97^ Electrostatic interactions govern IgG adsorption onto AuNPs, but the specifics of the binding depend on the pH of the solution. Decreasing the pH from 8.5 to 7.5 increases the number of positively charged surface regions on IgG molecules, altering their binding and preferred orientation onto the negatively charged surface of AuNPs. In addition, there is an increase in the antigen-binding capacity of IgG on AuNPs. Although IgG maintains its folded structure within this pH range, its localized regions may experience minor unfolding and rearrangements to reach its preferred orientation when adsorbed onto AuNPs. Modulating the pH of the environment allows for control over the charge distribution of IgG on AuNPs, its subsequent orientation, and antigen-binding accessibility, which is critical for its biological activity.

Variations in pH also impact the conformational changes of Lyz upon interacting with graphene oxide (GO).^98^ Under acidic conditions, electrostatic interactions govern Lyz–GO binding, leading to high-density adsorption of Lyz on GO. However, when the pH becomes greater than the pI of Lyz, the binding ability of Lyz and GO decreases as hydrophobic forces become more dominant. This is a result of Lyz undergoing slight conformational changes on the surface of GO, exposing its hydrophobic residues. Similar results are observed for Lyz and TiO_2_ surfaces at pH ranging from 3.6 to 10.8.^99^ Although the pH variations change the charge density of Lyz and TiO_2_, the intermolecular forces between Lyz and TiO_2_ are only affected by the pH-dependent effective diameter of Lyz, which determines the contact area.^99^

In a different approach, atomistic molecular dynamics (MD) simulations were utilized to study PC formation on dopamine-functionalized TiO_2_ NPs using two intracellular proteins overexpressed in cancer cells, nuclear protein poly(ADP-ribose) polymerase1 (PARP1) and heat shock protein 90 (HSP90).^100^ The simulations reveal that PARP1 corona residues contribute the most to the corona formation on cationic and neutral NPs under different pH conditions. These residues included β-turns, α-helices, and random-coil secondary structures, the percentage of which differs depending on the pH of the environment. However, random-coil, β-turns, and extended conformations were the essential motifs in the HSP90 PC formation on TiO_2_ NPs. In addition, both PARP1 and HSP90 have an increase in their random-coil structure and a decrease in their β-turn secondary structures in the presence of TiO_2_ NPs. The PARP1 contribution to the PC increases under less acidic intracellular pH conditions and cytosolic ionic strength, while the same ionic strength conditions decrease the HSP90 contribution.

Since PC formation depends heavily on the affinity of amino acid residues for NPs, pH-dependent amino acid speciation, especially for carboxylate- and amine-containing amino acids, also affects the adsorption of proteins onto the surface of TiO_2_ NPs.^101^ Increasing the pH from 2 to 9 results in higher adsorption of glycine (gly) and lysine (lys) amino acids, whereas it decreases glutamic acid (glu) adsorption. When the pH of the solution is close to its pI, serine (ser) adsorption to TiO_2_ NPs reaches its maximum.

The resistance of proteins within the PC to physiochemical fluctuations such as pH variations relates to their degree of hydrophobicity, β-sheets, α-helical structure, and amino acid content.^35^ Although pH variations cause the exchange of proteins with a lower affinity in the blood plasma PC of AgNPs, 47% of identified proteins in the PC retain their binding activity, and 60% of the persistent proteins maintain their abundance despite pH variations. The strong resistance of such proteins to physiochemical perturbation is attributed to their higher hydrophobicity, a greater number of β-sheets, and lower content of α-helices, which provides them with a highly stabilized structure and allows them to maintain their binding motifs under perturbed physiochemical conditions.

### c. Effect of protein concentration

During their journey *in vivo*, NPs experience unique biological fluids containing different amounts and types of proteins.^36^ For instance, when NPs extravasate from blood circulation, they are usually exposed to environments with lower amounts of proteins, such as interstitial fluid.^36,37^ Therefore, exploring the impact of molecular crowding on the evolution and conformational changes of PC is of utmost importance.^11,102^

The effect of molecular crowding has been demonstrated in cationic-ligand functionalized gold nanorods (AuNRs) and BSA.^103^ The adsorption of low protein-to-NP ratios leads to irreversible adsorption of a single or few BSA proteins on the surface of AuNRs, where many become unfolded. Changes in the secondary structure of adsorbed BSA cause AuNR aggregation through unfolded BSA–BSA interactions, which increases their cellular uptake to cancer cells. In contrast, incubation of AuNRs in a high physiological concentration of BSA leads to a stable monolayer of PC surrounding AuNRs under equilibrium conditions, which reduces their cellular uptake to cancer cells. Similar results are observed with PC formation on AuNPs from single proteins, including IgG, FBG, apolipoprotein A1 (ApoA1), and HSA, at varying concentrations.^39^ Under physiological conditions, a high protein : AuNP ratio leads to a stable and complete PC formation that sterically stabilizes AuNPs and prevents their aggregation. In contrast, a low protein : AuNP ratio causes significant aggregation of AuNPs. Additionally, when PC-AuNPs, formed at a high protein : AuNP ratio, shift from physiological conditions into an acidic tumor microenvironment, no further aggregation is detected. In contrast, PC-AuNPs formed at a low protein : AuNP ratio show signs of aggregation when entering an acidic tumor condition, except for PCs composed of HSA.

Contrary results are observed in cationic liposomes incubated in human plasma and diluted at different concentrations.^36^ Liposome–biomolecular corona (BC) complexes possess monomeric structures with the most significant negative zeta potential values at concentrations below 50 μg mL^−1^, while protein concentrations above 250 μg mL^−1^ lead to NP aggregation and less negative zeta potential. Although molecular crowding could undermine the stability of liposome–BC complexes, it did not affect the composition pattern of the BC across the concentration range. However, the impact of varying serum concentrations on PC composition and the resulting cellular interactions has been reported for SiO_2_ NPs.^104^ SiO_2_ NPs incubated in a low or a high concentration of serum lead to low-serum and high-serum corona–nanoparticle complexes (LC and HC, respectively). HC-NP uptake into HeLa cells is lower than LC-NPs, which is attributed to the differences in their corona compositions, particularly the abundance of histidine-rich glycoprotein (HRG) in HC-NPs. LC-NP uptake is primarily mediated by clathrin-mediated endocytosis and macropinocytosis mechanisms, while RNA interference analysis indicates that LDL receptors are involved in the uptake of HC-NPs.

FN conformational changes on the substrate and cellular interactions are also impacted by protein concentration.^105^ At high concentrations of FN on hydroxyapatite (HAp), FN has a fibrillar structure with end-on orientation and low cellular attachment to MG63 osteoblast-like cells. In contrast, low-concentration FN has an oblate ellipsoidal structure with side-on orientation, resulting in high cell adhesion. At low bulk concentrations, FN adsorbed on a hydrophobic polystyrene surface undergoes significant unfolding.^106^ However, protein–protein interactions and molecular packing reduce the unfolding of FN at high bulk concentrations. The addition of other proteins, such as HSA, affects the conformation of FN independent of protein concentration.^102^ When added simultaneously using a glass surface, HSA restricts the unfolding of FN by sterically blocking the surface before the adsorption of FN. This highlights the significance of exposure order in the ability of HSA to restrict the unfolding of FN, which is relevant for biomaterials exposed to environments containing different proteins. When HSA is added sequentially, its constraining effect on the unfolding of FN is insignificant, implying that introducing HSA when FN is already adsorbed on the glass would only slightly restrict its unfolding by displacing it or inducing its refolding.

The conformation of FN on AuNPs also depends on the protein concentration of the medium, which can be determined by the accessibility of FRET-labelled FN on the surface of AuNPs.^86^ PC-coated AuNPs incubated in 0.1% human plasma have more surface available for FN interactions than PC-coated AuNPs formed in 100% human plasma. When FN forms a hard corona by directly accessing the surface of bare AuNPs, it undergoes significant unfolding. However, for a corona-coated AuNP, FN accesses the corona *via* protein–protein interactions and maintains its original conformation; though some FN do undergo slight conformational changes due to protein exchange to access the AuNP surface. The results suggest that protein concentration in a medium can determine NP surface coverage as well as the extent to which proteins associated with the PC undergo conformational changes.

Protein molecules that have already been absorbed on a surface can either facilitate or prevent the adsorption of additional protein molecules, which is called a positive or negative cooperative effect, respectively.^29^ Still, proteins can adsorb onto the substrate *via* a non-cooperative effect if some regions are left unoccupied by proteins.^28^ The impact of cooperative interactions between proteins on the evolution of the PC is well documented.^26,107,108^ For example, the kinetics of the adsorption process for the hard PC formation is slower than the formation of the soft PC, suggesting the significance of cooperative effects.^108^ Stiff and hydrophobic annealed poly-l-lysine (PLL)/sodium alginate (Alg) polyelectrolyte multilayers (PEMs) with a high number of negative moieties lead to a cooperative interaction between BSA and FN in the PC.^109^ This results in the tight adsorption of both proteins onto the substrate, decreasing exchangeability and increasing adhesion to C2C12 myoblast cells. The increased cell adhesion on the hydrophobic surface is attributed to changes in the secondary structure of FN and likely changes in its 3D structure that exposes its RGD motif for integrin binding. In contrast, in the case of annealed chitosan (Chi)/hyaluronic acid (HA) PEMs with a hydrophilic surface and slight negative charge, non-cooperative interactions between proteins are observed, resulting in decreased adsorption of FN with a significantly high exchangeability and reduced cell adhesion. This underscores the significance of a substrate's nature in the cooperative interaction of proteins within the PC.

The important effect of protein exposure order is demonstrated in AuNPs incubated in BSA followed by collagen.^38^ Mimicking the exposure condition of intravenously injected NPs, BSA adsorption on AuNPs is reversible and could be replaced by collagen proteins that have a stronger affinity for the surface of AuNPs. In contrast, pre-incubation of AuNPs with low concentrations of collagen leads to a hard collagen corona formation that precludes BSA adsorption on the surface of AuNPs. This suggests that developing a pre-formed collagen PC on AuNPs could avoid further protein adsorption before reaching the target site. This is important, for example, in NP-mediated delivery of chemotherapeutics to tumors, as blood proteins can redirect NPs away from cancer cells. Still, *in vivo* studies are needed as blood contains a complex milieu of proteins. The importance of protein ordering has also been showcased in the interaction of alpha-synuclein (αS) and SiO_2_ NPs.^110^ In a protein-free solution, the entire polypeptide chain of αS interacts with SiO_2_. However, introducing Tau, extra αS, and BSA in solution results in complete, partial, and no detachment of αS from the surface of the SiO_2_ NPs, respectively.

For the successful clinical translation of nanotherapeutics in oncology, it is critical to examine the exchange and influence of tumor microenvironment enzymes on the PC. Interestingly, matrix metalloproteinases (MMPs), proteases overexpressed in cancer cells, can rearrange, exchange, and degrade the pre-formed PC of AuNPs.^111^ Furthermore, its effects on the PC of AuNPs differ depending on the nature of the corona. Certain tightly adsorbed proteins do not exchange or break down, which could be due to the specific conformation of the proteins on NPs, masking epitopes that MMPs can digest. This necessitates additional investigation into the relationship between protease activity and PC structural changes.

Different diseases may vary the hard PC composition on NPs, often known as “specific-disease PC”, which is most likely related to changes in the concentration and structure of plasma proteins regulated by diseases.^112,113^ PC formation from the plasma of non-small cell lung cancer (NSCLC) sources substantially reduces the cellular uptake of Tf-modified polyethylene glycol (PEG)-NPs (Tf-NPs), which is not the case for the PC from healthy sources.^114^ Proteomic analysis reveals that the PC of NSCLC-derived NPs is enriched not only with opsonins, which trigger subsequent immune responses but also with wound healing and tissue repair-related proteins, which causes protein aggregation and thus prevents Tf–TfR binding. However, a higher concentration of alpha-2-macroglobulin (A2M) is found in the PC of NPs incubated in healthy sources and is associated with uptake *via* endocytosis into A549 cells. As a result, pre-coating Tf-NPs with PC from healthy mouse models improves the tumor-targeting capacity of paclitaxel-loaded Tf-NPs in mice with NSCLC. This suggests that the use of pre-coated NPs with PCs derived from physiologically healthy sources is a promising cancer treatment method. Additionally, future research must consider critical differences in the microenvironment of healthy and pathogenic tissues, as well as their respective inherent heterogeneity on PC formation and structure.

### d. Effect of protein size and glycosylation

The size of proteins in biological medium is an essential yet overlooked factor in the interactions of NPs and proteins. Classification of proteins based on their properties such as size, structural stability, and composition has been previously proposed.^28^ In this classification, small and rigid proteins such as lysozyme, β-lactoglobulin, or α-chymotrypsin (ChT) are referred to as ‘hard’ proteins, implying a low tendency for structural changes upon surface adsorption. Abundant plasma proteins such as albumin, Tf, immunoglobulins, *etc.*, with a predisposition for conformational reorientations upon adsorption on surfaces, are considered intermediate-sized proteins, while high molecular weight proteins include polymer-like lipoproteins and glycoproteins whose behavior is essentially dominated by the content of lipids or glycans.

A recent study has considered protein size as the only variable affecting NP–protein interactions.^115^ Their results suggest that SiO_2_ NPs bind better to larger hemoproteins. Interestingly, at pH 7, all proteins have a decreased affinity than at pH 6. Enthalpic electrostatic interactions govern the adsorption of smaller proteins onto NPs. Smaller-sized proteins form homogenous PC monolayers surrounding the NPs, which lead to subtle changes in their tertiary structure, while preserving their shape and function. However, larger protein adsorption is entropy-driven and leads to the formation of an incomplete PC with no protein structural changes. Despite the absence of structural changes for larger proteins, their adsorption results in NP aggregation, especially at pH 6. This stems from larger proteins bridging with several NPs, leading to aggregation.

In the same vein, the impact of protein glycosylation on PC formation and NP–cell interactions is another relevant factor, especially as most human plasma proteins are glycosylated, and various disorders can alter the glycan profile of proteins.^116–118^ Glycosylation of the PC has a pivotal role in maintaining the colloidal stability of SiO_2_ NPs and their cellular interactions.^116^ Although enzyme deglycosylation of PCs leads to partial removal of glycans on the surface of the PC, it exposes otherwise hidden glycans within the complete PC. Deglycosylation expedites non-selective hydrophobic and electrostatic interactions between proteins, thereby decreasing the colloidal stability of SiO_2_ NPs. It also promotes cellular adhesion and uptake of SiO_2_ NPs and induces a pro-inflammatory milieu by THP-1 differentiated macrophages, underscoring the significance of glycosylation in immunological interactions. Similarly, deglycosylation of very-low-density lipoprotein (vLDL) and clusterin coronas significantly boosts cellular uptake by mice macrophages.^119^ In contrast, deglycosylation considerably reduces the cellular uptake of Apo AI and high-density lipoprotein (HDL) coronas.

The accessibility of glycans in the PC of citrate AuNPs influences the interactions with lectins, glycan-binding proteins, in biological environments.^120^ Deglycosylation of the PC results in no or very minimal interactions with lectins, implying that glycosylation of a PC is essential for efficient lectin binding and subsequent NP–cellular interactions. The glycan profile of the PCs has been exploited with SiO_2_ NPs to distinguish lung cancer patients from healthy groups.^118^ Plasma FBG is enriched using SiO_2_ NPs with a specific plasma : NP ratio to the point where its proteomic and glycomic “fingerprints” could be reliably traced to distinguish lung cancer patients from the healthy groups. The degree of FBG sialylation, a type of *N*-glycosylation, impacts its solubility and blood clotting process. Higher degrees of sialylation in FBG result in lower rates of fibrin polymerization and thinner fibers. Global sialylation is increased in the full plasma analysis, while FBG can undergo desialylation in the FBG-enriched corona, implying higher blood clotting incidents in lung cancer patients. Since protein glycosylation has direct implications for the composition of the PC and the destination of NPs, future studies should consider other post-translated modifications, such as phosphorylation, lipidation, hydroxylation, and methylation, of proteins within the context of PC and NPs.

Along with PC formation, protein glycosylation directly impacts PC conformational changes.^117^ Glycosylated human Tf and its non-glycosylated recombinant form (ngTf) show different secondary structural transitions when incubated with silver and AuNPs of various sizes, shapes, and surface functionalizations. A decrease in α-helix and β-sheet contents of Tf is correlated with higher binding affinity to NPs, while an increase in α-helix and a drop in the β-sheet structure of ngTf are attributed to a stronger binding affinity to NPs. Furthermore, the amount of Tf and ngTf in the PC did not correspond with their binding affinities for glutathione silver NPs (GSH-AgNPs), PVP-coated AgNPs, CIT-coated AuNPs, and PEG-AgNPs. This discrepancy can be attributed to the different degrees of protein conformational changes at the bio-nano interface. This demonstrates that the glycosylation mode of proteins impacts binding strength and changes in the secondary structure of proteins adsorbed on NPs. Additionally, it is likely that glycosylation levels of Tf in different diseases can be exploited for diagnostic purposes.

### e. Effect of temperature and shear flow

The average human body temperature varies from 35.8 to 37.2 °C and changes depending on the area of the body and overall condition.^121,122^ The physiologically relevant temperature variations between 37 and 41 °C affect the degree of protein coverage, the composition of the PC, and the cellular uptake of NPs.^121^ Elevated temperatures can induce irreversible protein conformational changes and denaturation in specific proteins.^123–125^ Incubation of PEGylated polystyrene NPs with heat-inactivated serum and plasma increases their macrophage uptake. Additionally, heat inactivation of serum and plasma dramatically decreases the amount of clusterin in the PC, while the amount of Apo AI remains high in the PC of NPs.^126^ Heat inactivation of serum and plasma leads to the enrichment of immunoglobulins and acute phase proteins in the PC of NPs. Still, their amount is negligible when NPs are incubated with native serum and plasma. In contrast to clusterin that has a melting point (*T*_m_) of 46 °C, Apo AI possesses a higher *T*_m_ (58 °C) and refolds upon cooling. Therefore, the initial incubation condition determines the structure and affinity of proteins for NPs, and this controls the PC composition as well as cellular uptake of NPs. The PC of AuNPs is stabilized by the covalent bonds of thio-proteins such as β-lactoglobulin, which comprise the hard PC, or the electrostatic interactions of non-thio-proteins such as myoglobin, which comprise the soft PC.^127^ Temperature has a major role, specifically in the adsorption of the thio-proteins. Although increasing the temperature decreases the binding forces and the number of adsorbed β-lactoglobulin, it results in a faster thiol covalent bond formation in the β-lactoglobulin PC of AuNPs.

While increasing the temperature denatures BSA in solution, the structure of BSA adsorbed on TiO_2_ NPs remains intact with increasing temperature, suggesting that the conformational changes of BSA upon adsorption on NPs enhance BSA thermostability.^128^ In contrast, increasing the temperature induces the same secondary structural changes of FBG when FBG is in solution and adsorbed on NPs. Hence, the thermostability of adsorbed proteins depends on their initial interaction with NPs and the type of conformational change induced by NPs, which can be different for each protein. The heat treatment of plant proteins such as glutenin, soy protein isolate, gliadin, and zein, affects their binding affinity to TiO_2_ NPs as well as their mass in the hard and soft PCs, depending on the type of protein.^129^ For example, a high-temperature treatment (100 °C) decreases the binding affinity of glutenin, a temperature-sensitive protein, to TiO_2_ NPs. At the same time, the high temperature has a much lower effect on gliadin due to its heat-resistant nature. The elevated temperature decreases the mass of glutenin in the soft layer, while the opposite is the case for zein, gliadin, and soy protein isolate. This suggests that the heat-induced unfolding and aggregation of zein, gliadin and soy protein isolate increase their adsorption. However, the unfolding and aggregation of glutenin leads to its dissociation, decreasing its adsorption.

Shear flow is an essential factor affecting the PC of NPs injected intravenously, yet it is mainly overlooked in most studies. The flow rates of the circulatory system range from relatively slow capillary speeds (0.085 cm s^−1^) to faster artery flow (10 cm s^−1^), with maximum velocities of 60 cm s^−1^ in the aorta.^130–134^ The PC composition of liposomes incubated with circulating FBS differs from the PC formed under static conditions.^135^ Circulating conditions contain more apolipoproteins and acute phase proteins while it is less enriched in complementary proteins. In contrast, the PC of PEGylated liposomes formed in a circulating flow is more negatively charged and contains a wider variety of proteins than its counterpart formed under a static fluid.^136^ Of note, the alterations in the composition of the PC of lipid NPs under a dynamic flow depend on both the time of exposure and the surface chemistry of the NPs.^137^

The shear stress generated by blood flow can change the structure of proteins.^138^ These changes in response to flow can differ depending on the type of protein, especially their intrinsic characteristics as well as their solution properties. The conformation and structural changes of proteins in response to flow can have implications for their binding affinity to NPs and their biological function. For example, the PC of polystyrene NPs forming in FBS under flow contains a greater concentration of proteins, especially plasminogen.^130^ Moreover, the PC formed under flow decreases the cellular binding of polystyrene NPs. Under a flow of 8.5 cm s^−1^ and in the absence of polystyrene NPs, plasminogen undergoes significant secondary structural changes, losing its ordered structure. However, the secondary structure of BSA in response to flow remains unchanged. This suggests that conformational changes of plasminogen in response to flow lead to its greater adsorption onto the surface of NPs, which can also impact its biological activity.

## 3. Role of nanoparticles in protein corona conformation

In addition to the biological factors discussed in the previous section, the intrinsic properties of NPs, including core composition, charge, curvature, shape, size, mechanical properties (*e.g.*, stiffness), and surface chemistry, have a major role in determining the composition and architecture of PCs. For instance, polyethylene glycol (PEG), a highly hydrophilic polymer, has been used to reduce protein adsorption and PC formation, and thus lengthen NP blood circulation, which further affects cellular uptake.^136,139,140^ However, a recent study has demonstrated that apolipoproteins, known to give nanomaterials stealth properties by reducing mononuclear phagocyte system uptake, are enriched in the HC of liposomes regardless of PEGylation, and they are preferentially enriched in the SC of the PEGylated liposomes.^141^ Given the non-biodegradable nature of PEG, there has been evidence of PEG accumulation as well as uncontrolled oxidative degradation into toxic products, which has inspired the use of polymers such as polyphosphoesters (PPEs) that do not pose the risk of accumulation.^21,142^ More details regarding polymers with stealth properties can be found in a review by Schöttler *et al.*^21^

The impact of charge and hydrophobicity of protein-based NPs on PC formation in FBS and macrophage uptake was examined by developing negatively charged BSA, cationic albumin (cBSA), and negatively charged ovalbumin (OVA).^143^ The following trend for the relative PC intensity, or PC mass, of the NPs was observed, BSA < OVA < cBSA. The findings reveal that OVA had more hydrophobic areas on the surface, whereas BSA had more internal hydrophobic sections. This suggests that surfaces with more hydrophobic areas adsorb more proteins. Each NP had a unique corona pattern, confirming the impact of the NP's surface property on the adsorption of specific proteins. Also, it is shown that the specific proteins in the PC of each NP regulate its recognition and uptake by macrophages.

PCs rapidly form on different-sized carboxylated polystyrene NPs (COOH-PS NPs; 26 nm, 80 nm, 200 nm) in mouse serum (MS).^144^ Gel electrophoresis results demonstrate that while 80 nm COOH-PS NPs had the most intense PC profile, the 26 nm NPs had the smallest PC. On 200 and 80 nm COOH-PS NPs, the identified bands corresponding to protein APOE and metalloproteinase inhibitor 3 (TIMP3) display a time-dependent profile that became weaker after 24 hours of incubation, while APO A-I protein intensity increased over time. Likewise, an increase in clusterin intensity, known to prevent macrophage uptake, was observed for the 80 nm COOH-PS NP. After 1 h of incubation, 80 nm COOH-PS NPs adsorbed most of the different proteins. However, the 200 nm COOH-PS adsorbed more myosin-9 and APO A-I proteins, whereas 26 nm COOH-PS NPs adsorbed fewer proteins than others. Proteomic analysis reveals a unique PC signature for different-sized NPs. To investigate the relationship between the mechanical properties of nanocapsules (softness & stiffness), PC composition, and subsequent macrophage uptake, oil-core silica shell nanocapsules modified with PEG with different Young's moduli (704 kPa, 25 MPa, 459 MPa to 9.7 GPa) were used.^145^ The total amount of proteins adsorbed onto the nanocapsules decreases as the stiffness of the nanocapsules increase, and each nanocapsule with stiffness ranging from 704 kPa to 9.7 GPa indicates a unique corona composition. Complementary and immunoglobulin proteins, which are essential in the immunological response and phagocytosis, were abundant in the PC of the stiffest nanocapsules, whereas apolipoprotein was less prevalent than in softer groups, which corresponds with their higher macrophage uptake compared to softer particles. Moreover, less macrophage uptake is observed for nanocapsules with PCs than those without PCs, regardless of their stiffness. Accordingly, PC formation minimizes the macrophage uptake of silica nanocapsules, and softer nanocapsules with more adsorbed proteins have less macrophage uptake. A more detailed review of the impact of NP characteristics on PC formation and composition can be found elsewhere.^19^

Since this review paper mainly focuses on conformational changes of proteins associated with the PC, we narrow down the topic and mainly focus on four types of NPs with extensive utility for biomedical applications, including gold NPs, silica NPs, iron oxide NPs, and quantum dots, to investigate their impact on the conformation and orientation of adsorbed proteins, as well as subsequent biological responses.

### a. Gold nanoparticles

When reduced to sub-100 nm structures, gold exhibits a variety of new characteristics that distinguish it from bulk gold.^146^ AuNPs have been used for a variety of biomedical applications due to their ease of preparation and surface modification, as well as their optical properties. Their stable and relatively inert nature in biological systems allow them to be biocompatible *in vivo*.^146–150^ Gold forms Au-thiol covalent bonds with proteins due to its high affinity for thiols.^127,151^ Also, it interacts with proteins *via* electrostatic forces. Given the extensive use of AuNPs for biomedical purposes, a growing body of studies has investigated the interaction of AuNPs with proteins as it relates to the fate of AuNPs as well as their *in vivo* toxicity.

Insulin fibril formation during manufacturing, storage, and following infusion or repeated injection into patients with insulin-dependent diabetes mellitus has been a major concern. In this regard, a recent study demonstrates how AuNPs coated with branched biopolymers such as dextran-40 and dextran-10 or linear biopolymers, including dextrin and chitosan, affect human insulin amyloid fibrillation in different manners.^152^ Linear biopolymer-coated AuNPs are the most effective inhibitors of insulin fibrillar formation due to their stable nature and strong interaction with insulin monomers, resulting in a stable AuNP-PC and suppressing the secondary structural changes of insulin from α-helix to β-sheet. However, AuNPs coated with branched biopolymers self-aggregate and have a weak interaction with insulin monomers, resulting in PC aggregates with a lower inhibitory effect on insulin fibrillation. Furthermore, AuNP-insulin amyloid fibrils and all types of biopolymer-coated AuNPs have lower toxicity towards pancreatic and HEK cells than pure insulin amyloid fibrils, suggesting dextrin-and chitosan-AuNPs as therapeutic delivery systems to inhibit insulin aggregation.

The ability of cytochrome (cyt c) to initiate cell apoptosis has sparked interest in its delivery to tumor cells as a therapeutic protein. A recent study showcased the impact of the physiochemical properties of the AuNP delivery system on cyt c conformation, orientation, and subsequent biological activity *in vivo*. Anionic ligands on AuNPs disrupt the tertiary structure of cyt c, while cationic and neutral ligands maintain cyt c structure.^153^ Furthermore, the surface charge of AuNPs determines the apoptotic and peroxidase activity of cyt c, which is governed by the accessibility of its heme ring in relation to its structure and orientation on AuNPs. Secondary structural changes of HSA adsorbed onto AuNPs depend on the curvature of the AuNPs, the type of surface ligands, and the pH of the medium.^154^ A high curvature correlates with smaller deformation in adsorbed proteins. Neutral PEG-OMe-AuNPs do not affect HSA structure. However, positively charged PEG-NH_2_-AuNPs cause significant conformational changes in the HSA PC regardless of the pH condition, and the resulting particles have the least cellular uptake and cytotoxicity in MDA-MB-231 human breast cancer cells, suggesting that unfolded HSA on PEG-NH_2_-AuNPs is unlikely to trigger any receptor-mediated phagocytosis process. Therefore, NP-induced conformational changes in the PC regulate not only cellular uptake but also the cytotoxicity of NPs.

AuNCs functionalized with dihydrolipoic acid (DHLA) and glutathione (GSH) impact the enzymatic activity of Lyz due to the dominant hydrophobic interactions, although both AuNCs initially interact with Lyz *via* electrostatic attractions.^155^ Each DHLA-capped AuNC adsorbs only one Lyz and induces secondary structural changes, inhibiting the enzymatic activity of Lyz. In contrast, hydrogen bonding and van der Waals forces governed GSH-capped AuNC interactions with Lyz, with each GSH-capped AuNC attaching to 3–4 Lyz and minimally inducing conformational changes in the protein. A similar inhibitory effect is observed for DHLA-AuNCs on the enzymatic activity of ChT, whereas GSH-AuNCs had no effect on ChT.^156^

Ligand adsorption modes, such as physisorption or chemisorption, impact the conformational changes of blood proteins. For example, physiosorbed citrate ligands on AuNPs gradually shed as they approached proteins, thereby exposing the bare surface of AuNPs.^31^ As a result, proteins on citrate-AuNPs undergo greater conformational changes than on chemisorbed GSH-AuNPs, owing to the high interfacial energy of AuNPs. A decrease in the internal energy of the proteins causes protein structure changes, with an increase in hydrogen bond formation. This results in a decrease in α-helical content and an increase in β-sheet content to compensate for the high interfacial energy. This supports the notion that conformational rearrangement of proteins can occur either intermolecularly or intramolecularly. Additionally, NPs functionalized with physiosorbed targeting molecules are likely to lose their targeting activity *via* ligand decomplexation, thus inducing significant, and often unwanted, conformational changes in the PC.

Interestingly, folic acid functionalized AuNPs (FA-AuNPs) and gold shelled Fe_3_O_4_ NPs (AuFeNPs) do not alter the secondary structure of HSA and Hb.^157^ Charged FA-AuNPs interact with both proteins *via* electrostatic interactions, whereas neutral AuFeNPs interactions with proteins is protein-dependent. For instance, AuFeNPs interact with Hb mainly *via* hydrophobic forces, but hydrogen bonding governs its interactions with HSA. In addition, FA-AuNPs–protein complexes are more stable than that of AuFeNPs, suggesting that the charge functionalization of AuNPs is an effective way of controlling protein–NP complexation. Still, this phenomenon can be protein-dependent, as demonstrated by the interaction between AuNPs and collagen. Due to the triple-helical conformation of collagen, the protein remains intact after interacting with negatively and positively charged functionalized AuNPs.^158^ In fact, dendrimer-functionalized AuNPs improve cell viability in HaCaT human epidermal keratinocyte cells, which can be exploited for collagen stabilization in tissue engineering and cosmetic applications.

**Fig. 4:**
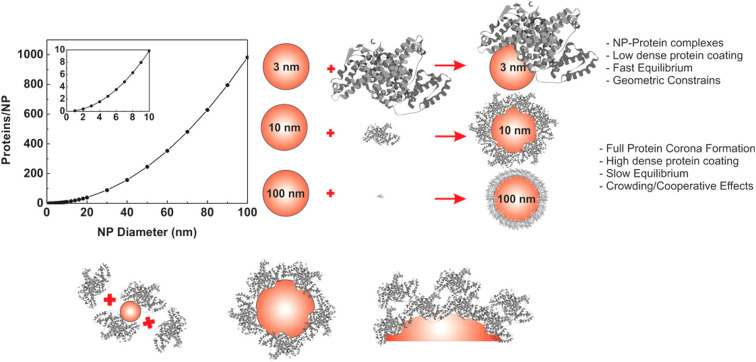
Size-dependent formation of the hard PC on AuNPs. The graph on the left represents the number of proteins that can form a single monolayer on the surface of the NP. Reprinted with permission from ref. 14. Copyright 2017. American Chemical Society.

The evolution of hard PC formation on AuNPs depends on the size of the particle.^14^ As the size of AuNPs increases, the following transition regime of PC has been documented (Fig. 4). First, AuNPs complex with proteins to form an incomplete PC. Then, a near-single dense PC layer forms. Finally, a multilayer PC develops. Similar results are observed for the interaction of HSA and Tf with DHLA-AuNCs with a core size of 2 nm.^159^ Each HSA and Tf molecule attaches to eight and seven DHLA-AuNCs, respectively, suggesting the formation of a protein complex rather than a PC. Additionally, the DHLA-AuNCs only slightly modulate the secondary structure of the proteins, showcasing the biocompatibility of DHLA-AuNCs.

The binding affinity and conformational changes of proteins on cationic AuNCs depend on the physicochemical properties of each protein, such as their molecular weight (MW) and isoelectric point (pI).^160^ BSA likely forms an AuNCs–protein complex, while Lyz and myoglobin tend to form a PC layer. The interaction between BSA and myoglobin with AuNCs decreases the α-helix structure of the proteins, while increasing their β-sheet content. However, the secondary structure of Lyz remains almost intact, which correlates with its minimal affinity for AuNCs. Moreover, the adsorption of proteins with sufficient binding affinity for AuNCs significantly reduces their toxicity toward HeLa cells.

Intrinsically stabilized proteins such as GB3, a small immunoglobulin binding domain from *Staphylococcus aureus*, and bovine carbonic anhydrase (BCA), an enzyme responsible for converting carbon dioxide to carbonic acid and bicarbonate, are likely to form a single layer of compact proteins consistent with their globular conformation on the surface of citrate-coated AuNPs regardless of the size of AuNPs.^161^ Despite the compact globular conformation of intrinsically stabilized proteins on AuNPs, slight secondary structural perturbations would likely occur on the surface of AuNPs. In contrast, the Drosophila drkN SH3 domain, a small, intrinsically unstable domain, adsorbs in a greater packing density and substantially unfolds when bound to AuNP surfaces. This emphasizes the importance of the intrinsic stability of adsorbed proteins in their adsorption behavior. There are several explanations for the aberrant adsorption behavior of the drkN SH3 domain to AuNPs. It is likely that its unfolded state is more favored than the folded state. Hence, the unstructured drkN SH3 domain binds directly to the AuNPs. Another explanation is that the folded state might be preferred for binding to the NP surface. In this case, the protein is initially globular, but it would undergo deformation after adsorption and result in aberrant binding. However, it is possible that a combination of the two possibilities could be the explanation.

The amount of protein required to establish a moderately stable PC on AuNPs differs depending on the protein.^162^ The interaction between AuNPs and proteins such as trypsin, pepsin, γ-globulin, and hemoglobin is governed by hydrophobic interaction, while van der Waals forces and hydrogen bonding dominate lysozyme–AuNP interactions. The degree of AuNP-induced secondary structural changes of proteins varies by the type of protein, where AuNPs reduce the activity of lysozyme, trypsin, and pepsin. Trypsin undergoes substantial AuNP-induced secondary structural changes, while AuNPs slightly affect the secondary structure of FBG.^149^ Irregular-shaped AuNPs, such as nanorods (AuNRs) and nanostars (AuNSs), induce more substantial secondary structural changes in FBG and trypsin than nanospheres (AuNSPs).^149^ FBG interaction with AuNSPs leads to the formation of a stable PC without triggering AuNSP aggregation; however, the introduction of trypsin causes considerable aggregation of AuNSPs. Thus, the morphology of AuNPs, as well as the properties of adsorbed proteins, affects PC formation and conformation as well as protein-triggered NP aggregation. The morphology of AuNPs and the type of adsorbed protein affect the extent of Lyz and ChT adsorption on AuNPs and their subsequent structural changes, as well as protein-triggered AuNP aggregation.^163^ Branched-shaped AuNPs adsorb HSA molecules in different orientations due to the multi-oriented tips of the AuNPs, while HSA molecules adsorb onto spherical-shaped AuNPs in one direction.^164^ Moreover, the thickness of the PC depends on the shape of the AuNP and is estimated to be smaller for spherical-shaped AuNPs than branched-shaped AuNPs.

NPs with different core compositions and curvatures have distinct impacts on the enzymatic activity of proteins. For example, AuNPs that have a size of 5 nm attach to the heavy chain of coagulation factor XII (FXII), one of the essential zymogens in the blood coagulation process, and adsorb FXII in a standing-up fashion without causing any subsequent structural alterations or activation of the protein.^165^ However, silica and silver NPs adsorb FXII in a lying-down position and induce conformational changes in FXII, causing the cleavage and activation of the zymogen. Investigating the effect of NPs on the enzymatic activity of α-FXIIa reveals that AuNPs and silver NPs that are 5 nm in diameter cause non-competitive and competitive inhibition of α-FXIIa enzymatic activity, respectively. In contrast, silica and silver NPs that are 20 nm in diameter promote α-FXIIa enzymatic activity by inducing favorable conformational changes in the proteins, suggesting that the relatively low curvature of the NPs improves the enzymatic activity.

The chirality of AuNCs also impacts the biological behavior of proteins, including FXIII.^166^d-Cysteine-coated AuNC (d-AuNCs) have a weak binding affinity for FXIII but induce considerable conformational changes and aggregation in FXIII, where they activate FXIII for cleavage. In contrast, l-cysteine-coated AuNCs (l-AuNCs) display a strong binding affinity for FXIII and restrict its conformational changes and aggregation. d-AuNC only increases the enzymatic activity of α-FXIIa, while l-AuNC improves both its enzymatic activity and efficiency. Similarly, the adsorption of BSA on l- and dchiral surfaces of AuNPs results in distinct orientations, affinity, exposed charges, and thermodynamics.^150^ Despite forming a BSA PC monolayer on the chiral surfaces of AuNPs, the conformation of BSA remains intact with no AuNP-induced secondary structural changes.

DNA-templated Au nanoclusters (DNA-AuNCs) interact with HSA *via* van der Waals interactions and hydrogen bonding. DNA-AuNCs preferentially bind to HSA, quenching its intrinsic fluorescence and slightly altering its secondary structure. This reduces the biological activity of HSA, highlighting the potential toxicity of DNA-AuNCs.^167^ AuNP-induced protein aggregation at physiological pH results in the formation of protein–AuNP agglomerates accompanied by free large protein aggregates in solution.^168^ However, no AuNP–protein assembly is observed when AuNPs are pre-coated with a high concentration of PEG, underscoring the biosafety risks associated with using unfunctionalized and partially functionalized AuNPs.

It was recently discovered that both the omicron and alpha spike proteins of SARS-CoV-2 have a higher binding affinity for nano-gold colloids with diameters greater than 30 nm, but have a very low affinity for gold colloids with diameters less than 20 nm.^169^ This is attributed to the comparable size of spike proteins with gold colloids of smaller sizes. Changing the pH from 3 to 11 induces reversible gold colloid aggregates, which is not observed for gold colloids with diameters of 10, 15, and 20 nm. Under low pH conditions, the hard PC of the omicron spike protein undergoes conformational denaturation and is less resilient than alpha spike protein, indicating the susceptibility of omicron spike protein in an acidic intracellular environment.

### b. Silica nanoparticles

Silicon is the most abundant element on Earth, besides oxygen, and the most abundant mineral in the Earth's crust is crystalline silica in the form of quartz.^170^ Silica NPs have favorable properties – biocompatibility, biodegradability, high mechanical strength, and the ability to induce tissue repair – making them ideal candidates for biomedical applications such as drug and gene delivery, bioimaging, and biosensing.^171^ Silica NPs have a long record of governmental approval as they are frequently used in the cosmetic and food industries.^172–174^ The interaction of silica NPs with proteins can exert irreversible impacts on the structure of proteins and their functionality, thereby affecting NP–cell interactions.

Mesoporous SiO_2_ NPs are synthesized in different shapes by adjusting the concentration of surfactants, such as cetyltrimethylammonium bromide (CTAB), a cationic surfactant commonly used for the synthesis of mesoporous SiO_2_ NPs as well as ammonia, and tetraethyl orthosilicate (TEOS).^175,176^ HSA undergoes significant secondary structural changes on the surface of spherical and rod-shaped mesoporous SiO_2_ NPs with different pore scales, which improves the binding stability of HSA and the saturated adsorption capacity of the NPs.^176^ FBG on the surface of spherical-shaped mesoporous SiO_2_ NPs with small and large pore sizes bends to accommodate the surface curvature of NPs, losing some secondary structure and boosting saturated adsorption capacity. In contrast, due to the stiff structure of globulin, different-shaped mesoporous SiO_2_ NPs with varying pore sizes did not cause significant secondary structural disturbances. A more recent study found that the structural changes of PCs derived from bovine serum are more pronounced for rod-shaped mesoporous SiO_2_ NPs than spherical and faceted mesoporous SiO_2_ NPs.^177^ Spherical mesoporous SiO_2_ NPs adsorb a higher albumin content and form relatively homogenous hard and soft PCs, while rod-shaped and faceted mesoporous SiO_2_ NPs primarily develop weakly bound soft PCs, with a dendritic pattern for faceted-shaped mesoporous SiO_2_ NPs.

BSA and myoglobin undergo size-dependent conformational changes on SiO_2_ NPs. Both proteins show conformational changes on NPs larger than 150 nm. Myoglobin interacts with NPs in a mixed-mode manner, where its denaturation on the NPs is rationalized by an indirect influence of the curvature. However, BSA interacts with SiO_2_ NPs *via* hydrophobic interactions, where it takes longer to undergo conformational changes than myoglobin.^178^ Increasing the size of SiO_2_ NPs or decreasing the surface curvature allows Lyz to have a narrower orientation distribution and undergo greater conformational changes due to strong electrostatic interaction between Lyz and large SiO_2_ NPs.^179^ However, a larger surface curvature as well as a higher ionic strength of the solution changes the preferred orientation of Lyz from the “bottom end-on” to the “side-on” orientation, which is unfavorable for anchoring Lyz in an enzymatically preferred orientation. The interfacial hydration layer for SiO_2_ NPs of lower curvature is stronger and has ordered interfacial water molecules, whereas Lyz can easily disrupt the hydration layer of NPs with smaller sizes and adsorb. This suggests that the size-dependent conformation and orientation changes of Lyz on SiO_2_ NPs are related to the first hydration layer surrounding SiO_2_ NPs, rather than the direct contact area between Lyz and SiO_2_ NPs.

Amorphous SiO_2_ NPs selectively adsorb threonine protease Taspase1, and non-competitively inhibit its proteolytic activity. Taspase1–NP interactions neither change the secondary structure of Taspase1 nor disrupt its stability. Instead, the inhibitory effect of the NPs is explained by Taspase1 binding to the NPs as a single layer of the αβ-dimer, such that the negative surface of NPs obstructs the positively charged active site of the Taspase1.^180^ However, although hydrophobic forces primarily govern the interactions between SiO_2_ NPs and catalase, the enzyme still retains its native structure and activity.^33^

The interaction of SiO_2_ NPs with human tau protein, whose intracellular neurofibrillary tangles are involved in Alzheimer's disease, changes the intrinsically unfolded structure of tau to partially folded structures and amorphous aggregates, which is accompanied by an increase in its α-helix and β-sheet contents and a loss of its random coil structures.^181,182^ Intrinsically disordered proteins (IDPs), such as α-casein, Sic1, and α-synuclein, preserve their structural disorder when adsorbed onto SiO_2_ NPs, forming a hard PC.^183^ They undergo negligible protein-specific conformational changes, resulting in minor folding and stabilization *via* increased secondary structure content. This is compared to Lyz, which undergoes significant structural perturbation and loss of its helical segments in the hard PC of SiO_2_ NPs. Although NP-induced secondary structural changes of α-synuclein in the hard PC of SiO_2_ NPs increased its helical segments rather than β-sheet contents, SiO_2_ NPs promote α-synuclein aggregation in a concentration-dependent manner. This suggests that the soft PC layer and local protein concentration are likely to have a role in promoting amyloid aggregation.

The interaction of BSA, Hb, and FBG with SiO_2_ NPs does not significantly change the secondary structure of adsorbed FBG, while the secondary structure of BSA and Hb changes with the increasing amount of SiO_2_ NPs, which leads to the loosening and unfolding of their structures.^32^ Furthermore, in contrast to BSA and Hb, FBG adsorption on SiO_2_ NPs delays the autophagy-inducing activity in HUVECs cells by stabilizing SiO_2_ NPs, preventing their sedimentation and cellular internalization. However, the protective effect of FBG only works for non-phagocytic cells and is limited to 24 h.

Although the adsorption of porcine Hb purified in the oxygenated form (HbO_2_) on SiO_2_ NPs leads to a substantial secondary structure loss, adsorbed HbO_2_ maintains its heme group and tetrameric structure, increasing its oxygen binding affinity and lower cooperativity.^184^ In addition, the structural–functional changes of adsorbed HbO_2_ are fully reversible when HbO_2_ desorbs at pH 8.7. The adsorption of human Hb onto SiO_2_ NPs induces significant secondary and tertiary structural changes in Hb, causing heme displacement and degradation, resulting in the release of iron in a concentration-dependent manner.^185^ Investigating the interaction between hydrophilic and hydrophobic SiO_2_ NPs with bovine Hb (BHb) under physiological conditions demonstrates that BHb has a higher affinity for hydrophobic SiO_2_ NPs, where PC formation increases the size of hydrophobic SiO_2_ NPs more than hydrophilic SiO_2_ NPs.^186^ In addition, hydrophobic SiO_2_ NP-induced changes in BHb, such as conformational changes around the tyrosine residues, facilitate the degradation of its heme group and enhances the fluorescence intensity compared to hydrophilic SiO_2_ NPs, suggesting the structure-based toxicity of SiO_2_ NPs.

### c. Iron oxide nanoparticles

Iron and oxygen combine chemically to produce iron oxide compounds.^187^ Magnetite (Fe_3_O_4_), maghemite (γ-Fe_2_O_3_), and hematite (HM; α-Fe_2_O_3_) are the three most common forms of iron oxides in nature. Magnetic iron oxides have attracted tremendous attention due to their characteristics, such as superparamagnetism, high magnetic susceptibility, high coercivity, low toxicity, and biocompatibility.^188^ They are extensively used in controlled drug delivery, magnetic resonance imaging, tissue repair, magnetic sensors, magnetic fluid hyperthermia, magnetic separation, and biolabeling.^189^ Therefore, the interaction of iron oxide NPs and biomolecules, as well as the biological response they elicit, should be carefully examined.

**Fig. 5:**
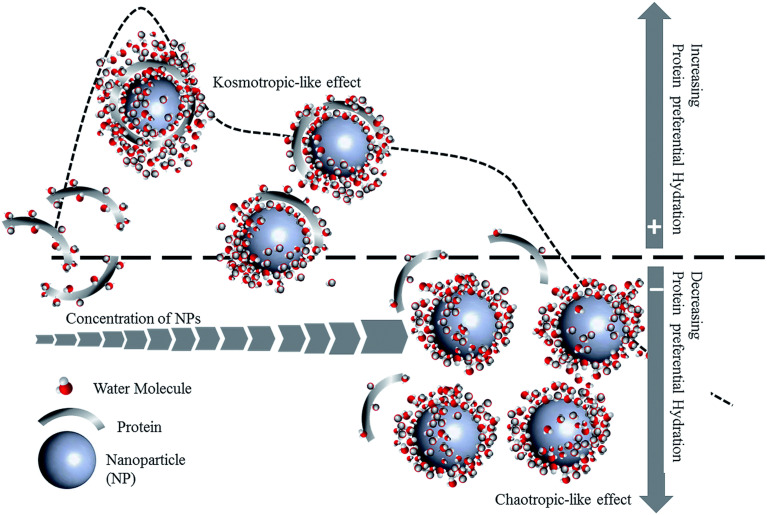
A hypothetical molecular mechanism of the protein preferential hydration and subsequent modification of protein stability by the kosmotropic-like and chaotropic-like effects of MNPs. Reprinted with permission from ref. 191. Copyright 2017. Royal Society of Chemistry.

The concentration dependence of NP–protein interactions, as well as the impacts on protein structure and activity, is an essential subject that needs more investigation.^190–192^ NPs affect the structure and biochemical equilibrium of proteins in solution by indirectly affecting the structure and dynamics of the hydration water shell surrounding the proteins.^190,191^ Protein adsorption on NPs alters the protein hydration configuration, resulting in changes in protein structure and activity. The lower concentration of MNPs has kosmotropic-like properties (Fig. 5), where their interaction with egg white Lyz leads to complete PC formation, increased protein preferential hydration and stabilized folded state of adsorbed Lyz molecules. On the contrary, increasing the concentration of MNPs reduces PC formation to such an extent that they are not surrounded by a PC at very high concentrations of MNPs. Thus, the uncovered surface of MNPs interferes with protein hydration, gradually disrupting protein structure stability in a chaotropic-like manner. However, coating MNPs with rosmarinic acid (RA), a natural polyphenol, and the amino acid arginine (Arg) increases the localized surface charge on MNPs as well as the hydration layer around Lyz, thereby stabilizing the protein's secondary and tertiary structures. This suggests that RA and Arg could be potential ligands for the modification of MNPs in a low concentration of proteins.

**Fig. 6:**
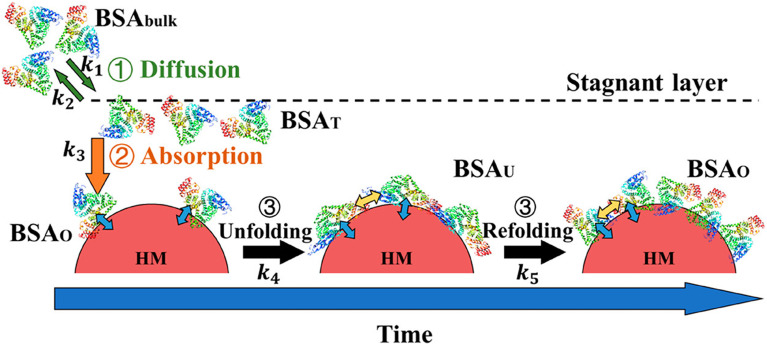
A schematic representation of the adsorption process of BSA onto HM NPs. BSA molecules penetrate the stagnant layer in the vicinity of the HM NP surface (BSA_T_) from the bulk solution (BSA_bulk_) at a rate constant of *k*_1_. The diffusion of BSA back to the bulk solution occurs at a rate constant of *k*_2_. BSA molecules might overcome the energy barrier of adsorption, which depends on various factors, such as the steric and electrostatic forces between proteins and particles as well as the osmotic repulsion from a water layer on the particle surface, and bind to the surface of HM NPs without conformational changes in a transient state at a rate constant of *k*_3_ (BSA_O_). Then, adsorbed BSA molecules undergo conformational changes within 30–60 min at a rate constant of *k*_4_ and change to an unfolded state (BSA_U_). However, when the surface coverage exceeds a threshold, BSA molecules refold at a rate constant of *k*_5_ (BSA_O_). It is worth mentioning that the values of *k*_4_ and *k*_5_ depend on the protein surface coverage changing over time. Reprinted with permission from ref. 194. Copyright 2019. American Chemical Society.

The formation of a BSA PC on magnetic iron oxide NPs (MIONPs) reduces BSA α-helicity while increasing its β structure. It suggests partial unfolding and aggregation of BSA on the surface of MIONPs, governed by hydrophobic interactions.^193^ The conformational changes of BSA on the surface of HM NPs depend on the initial concentration of BSA in bulk solution as well as the size of the NPs.^194^ Additionally, the conformational changes of adsorbed BSA are a dynamic process involving multiple states. The BSA–HM NP interactions are stronger for larger HM NPs, leading to faster conformational changes of adsorbed BSA due to loss of their α-helix content and inhibition of incoming BSA molecules' adsorption. This is a result of the open secondary structure of unfolded adsorbed BSA occupying more surface sites than its folded states. In addition, strong protein–HM NP interactions on larger HM NPs may counteract protein–protein interactions between neighboring proteins on the NP surface, preventing the refolding of adsorbed BSA. A kinetic model has been proposed for the adsorption and conformational changes of BSA on HM NPs (Fig. 6).

At pH 6, HM NPs induce protein-specific secondary structural changes in adsorbed BSA and beta-lactoglobulin (β-LG), followed by proteins returning to their solution-phase conformation after 90 minutes.^94^ However, phosphate pre-adsorption on HM NPs reduces protein surface coverage, slows the protein-specific kinetics of BSA and β-LG, and restricts secondary structural changes in proteins. The ability of phosphate to attenuate the HM NP-induced secondary structural changes of proteins can be attributed to its induction of steric constraints or bridging and ternary complex formation, suggesting phosphate as a potential agent in attenuating adsorbed protein denaturation, particularly at low surface coverage.

Along with phosphate, the role of counter ions can significantly impact the structure of proteins on the PC.^195,196^ For example, hen egg-white Lyz undergoes irreversible denaturation on the surface of iron oxide NPs (IONPs) functionalized with trisodium citrate (TSC) and sodium triphosphate (STP) containing Na^+^ counter ions.^195^ The denaturation is attributed to the diffusion of Na^+^ counter ions into the hydrophobic core of Lyz, thereby irreversibly unfolding its structure. The more pronounced denaturation of adsorbed hen egg white Lyz on the surface of STP-IONPs compared to TSC-IONPs can be attributed to the presence of more Na^+^ counter ions in the STP-IONPs dispersion. Moreover, the interaction of hen egg-white Lyz with pure STP without IONPs and PEG-coated IONPs did not disrupt its native structure. Counter ions also have a critical role in the inactivation of proteins by unfolding them on the surface of functionalized IONPs.^197^ In addition, counter ion size and charge affect the extent to which adsorbed hen egg-white Lyz undergoes unfolding on IONPs.^198^ For instance, compared to larger counter ions, smaller counter ions diffuse easier into the native structure of hen egg-white Lyz, thereby inducing more unfolding. However, although Mg^2+^ is smaller than Li^+^, it induces less unfolding due to its high charge attenuating its diffusion into hen egg-white Lyz.

Platelets have a significant role in hemostasis and serve as a catalytic surface for the coagulation cascade. Plasma proteins such as FBG and von Willebrand factor (vWF) are essential contributors to platelet aggregation and coagulation.^47,199,200^ The inhibition of platelet aggregation could affect wound healing and normal hemostasis in response to injury. In this regard, superparamagnetic iron oxide nanoparticles (SPIONs) can affect platelet functions depending on their concentrations, surface chemistry, and charges.^47^ Although positively charged SPIONs at 500 μg mL^−1^ induce platelet activation, SPIONs display a dose-dependent inhibitory effect on platelet aggregation regardless of their formulation. This is a result of SPION-induced conformational changes of FBG.

γ-Fe_2_O_3_ NPs also influence the secondary and tertiary structures of FBG.^188^ FBG can bind to the αIIbβ3 platelet receptor; however, SPION-triggered FBG structural changes impair its ability to bridge platelets, inhibiting subsequent platelet aggregation. Interestingly, contrary results have been reported for the interaction of FBG with γ-Fe_2_O_3_ NPs functionalized with citrate, dextran, and PEG coatings.^44^ FBG interaction with ligands or the surface of γ-Fe_2_O_3_ NPs did not induce any secondary structural changes. Moreover, FBG adsorption on citrate and dextran-coated NPs increases the binding affinity for integrin αIIbβ3 receptor of an artificial platelet membrane model, while PEG-coated NPs, which prevent FBG adsorption, had negligible interaction. Further studies are needed to fully understand the structure–function relationship between γ-Fe_2_O_3_ NPs and FBG interaction.

The interaction of superparamagnetic γ-Fe_2_O_3_ NPs with human blood plasma Tf containing different iron-saturation levels, including partially iron-saturated Tf and iron-free Tf (ApoTf), stabilizes the thermal stability of adsorbed Tf and ApoTf by increasing their melting points by 10 °C.^201^ The stabilizing effect of NPs on Tf and ApoTf likely stems from the adsorption of amino acids on the surface of γ-Fe_2_O_3_ NPs, which do not alter the protein structure, thus requiring higher energy to break the interactions. This may also be explained by the formation of a protein–NP conjugate that increases the iron saturation level of the protein. In contrast, the irreversible conformational changes of iron-saturated human Tf from a compact to an open jaw structure upon interaction with superparamagnetic iron oxide NPs have previously been reported and attributed to iron loss.^202^

### d. Quantum dot nanoparticles

Quantum dots (QDs) are nanometer-sized radiant semiconductor crystals with unique photochemical and photophysical properties owing to their small size and highly compact structure.^203,204^ Due to their bright fluorescence, narrow emission, broad UV excitation, and high photostability, QDs have great potential for optoelectronic and biomedical fields, such as photovoltaic cells, fluorescent dyes, drug delivery, and bioimaging for disease diagnosis and treatment.^205,206^ Nevertheless, the potential toxicity of QDs, which is primarily related to their composition and reactivity, is one of the main issues surrounding their application to living organisms.^45,207^ Based on their elemental composition, QDs can be distinguished into different groups, including cadmium-based QDs, cadmium-free metal QDs, and metal-free QDs containing novel polymer dots, black phosphorus QDs, and carbonaceous QDs such as carbon and graphene QDs.

Of note, the interaction of QDs with proteins can result in secondary structural changes and unfolding of proteins, impairing their functionality. Accordingly, a thorough investigation of QD–protein interactions and subsequent biological responses is necessary to determine the impact of PC on the toxicity of QDs. A detailed review of structural changes of proteins in the presence of different types of QDs and the biological consequences can be found elsewhere.^207^

HSA PC formation on water-soluble cadmium telluride (CdTe) QDs functionalized with thioglycolic acid (TGA-CdTe QDs) does not induce secondary structural changes in HSA and enhances the stability and photoluminescence of QDs.^208^ A kinetic and thermodynamic investigation has revealed that the interactions between CdTe-QDs and HSA involve transition-complex formation.^209^ The complex formation between CdTe QDs functionalized with mercaptopropionic acid (MPA-CdTe QDs) and HSA does not induce extensive conformational changes in HSA. The interaction of HSA with MPA-CdTe QDs was enthalpic and entropically favorable, while only entropy dominated the interaction of HSA with TGA-CdTe QDs. Contrary results have been observed for the interaction between molybdenum disulfide (MoS_2_) QDs and HSA, which is governed by hydrogen bonding and van der Waals force.^210^ MoS_2_ QDs gradually change HSA secondary structure from α-helical to β-turn, β-sheet, and random coils, leading to a looser HSA structure. Also, their interaction quenches HSA fluorescence and has an irreversible inhibitory effect on its fibrillation, confirmed by the disruption of disulfide bonds in the HSA network structure and the diminished hydrophobic environment. The findings suggest MoS_2_ QDs as a potential agent for treating protein misfolding-related diseases.

Along with the ligand functionalization, the chirality of QDs is an essential factor in controlling the interactions of nanomaterials and proteins.^211^ For instance, FRET-labeled HSA displays different binding affinity, adsorption orientation, and conformation on the surface of InP@ZnS QDs with different chirality, which could impact the exchange of HSA with other serum proteins as well as its cellular interactions.

Currently, there are concerns regarding the hemocompatibility of QDs due to their extensive use in biomedical applications, which have fueled research into the impact of QDs on homeostasis and the coagulation cascade. CdTe QDs have shown significant anti-coagulant properties related to the intrinsic coagulation pathway, which is independent of platelets and phospholipids.^212^ This could be attributed to the interaction of CdTe QDs with plasma and coagulation-related proteins. For instance, FBG, plasminogen, and prothrombin form bioconjugates with CdTe QDs and CdTe/ZnS QDs with varying affinities *via* hydrogen bonding and hydrophobic interactions.^65^ QD–protein interactions lengthen the activated partial thromboplastin time and prothrombin time. Additionally, these interactions alter the expression levels of coagulation and fibrinolytic factors, thereby changing the coagulation balance. QDs induce fluorescence quenching and conformational changes in the structure of proteins, where the latter varies depending on the type of protein. Molecular docking analysis has determined that the binding of QDs to active sites of proteins may encourage protein activation, interfering with hemostasis and fibrinolysis processes.

FBG and plasminogen have a high binding affinity for CdTe QDs.^213^ The FBG-CdTe QD complex decreases the β-sheet structure of FBG and increases its α-helix content, while the interaction between plasminogen and CdTe QDs changes its β-turns into β-sheet structures. The complex formation between thrombin and CdTe QDs decreases its α-helix and β-turn contents while significantly increasing its random coil content. CdTe QDs interact with Tf *via* hydrophobic interactions, forming a soft PC.^205^ The CdTe QD-Tf complex reduces CdTe release and cytotoxicity towards mouse primary kidney cells and changes the secondary and tertiary structures of Tf, loosening its polypeptide chains and decreasing its aggregation state. The conformational changes in Tf are caused by the surface of CdTe QDs rather than the released Cd.

The binding of carbon nanodots (CDs) to HSA and γ-globulins is governed by hydrophobic and van der Waals forces with little impact on the secondary structure of both proteins.^63^ In addition, CDs affect the transportation function of HSA and γ-globulins by changing the binding affinity of different drugs to the proteins. The binding between fluorescent CDs and HSA leads to a complex formation stabilized by hydrogen bonding and van der Waals interactions.^214^ Site I (subdomain IIA) of HSA primarily serves as the main binding site for CD. The complex formation induces concentration-dependent conformational changes in the secondary structure of HSA, causing HSA to become less compact and expose its hydrophobic cavities. Hence, the biological activity of HSA may decrease with higher CD concentration.

HSA forms a PC on CDs, where the structure of the protein is dependent on the surface modifications.^215^ The interaction between negatively charged PEG functionalized CDs and HSA does not induce significant secondary structural changes in HSA. In contrast, a high concentration of positively charged polyethyleneimine (PEI) functionalized CDs causes substantial secondary structural changes in HSA. The interaction between PEG-CDs and HSA is governed by hydrophobic and van der Waals forces. However, electrostatic forces are the driving interactions between HSA and PEI-CDs. Moreover, while the primary binding location of PEG-CDs is in site I of HSA, site II is the primary location for PEI-CDs. This emphasizes the importance of surface charge in CD–protein interactions.

Pyroglutamate (PGA) CDs are used as a fluorescent probe for peroxide detection in enzyme-based reaction systems.^216^ Studying the multifaceted interaction of CDs with coupled enzyme systems and HSA reveals that electrostatic interactions govern the binding of CDs with enzymes; altering their secondary structure by shifting most of their helix contents to β structures, exposing their tyrosine and tryptophan residues and reducing enzymatic activity.

CDs tightly bind to human Tf (hTf) *via* hydrophobic forces and electrostatic interactions, inducing structural changes in hTf.^217^ The conformational changes of hTf cause iron release from the hTf lobes, highlighting the subtle toxicological effect of CDs at the molecular level. The conformational changes in hTf could be due to the non-synergetic anion function of CDs, which bind allosterically to the hTf site and alter its structure. In contrast, there is a negligible effect of anionic CDs on the conformational changes of hTf.^218^ The interaction of near-infrared fluorescence light silicon QDs (NIR-SiQDs) with Tf and HSA leads to the formation of a hard PC, while FBG forms a soft PC on the surface of NIR-SiQDs.^219^ NIR-SiQDs induce changes in the secondary structure of the proteins. The α-helical structure of Tf decreases with increasing NIR-SiQDs concentration. However, at a NP concentration of 1.25 μg mL^−1^, the α-helical structure of Tf increases until it almost reaches its original form. A similar trend is observed for the secondary structural changes and recovery of FBG, which depend on the concentration of NIR-SiQDs. The secondary structure recovery of Tf and FBG in the presence of NIR-SiQDs implies that SiQDs are biocompatible for biomedical applications.

## 4. Leveraging protein corona conformation for therapeutic purposes

### a. Controlling protein conformation in assembled matrices

As stated in section 2, NPs come into contact with the ECM before reaching the cellular target site. The composition and structure of the PC can change as ECM proteins replace blood-circulating proteins, influencing NP–cellular interactions and downstream signaling. Moreover, the highly dense, fibrillar structure of the ECM is one of the major barriers to targeted delivery systems, which blocks the transportation of NPs into the target site. Understanding the variations in the composition and architecture of healthy and pathogenic ECMs is the first step toward engineering the ECM to improve targeted delivery. A detailed review of the most recent advances in targeting the ECM for therapeutic purposes can be found elsewhere.^220,221^ In this section, we proceed by focusing on an ECM protein FN, as it is the first provisional ECM protein assembled and dysregulated during disease progression, and we further provide insights on how the matrix-derived PC of NPs could be exploited for therapeutic purposes.

The extracellular matrix (ECM) is a cell-secreted dynamic fibrillar network that controls cellular activities under physiological conditions such as embryonic development and wound repair, and disease conditions, such as cancer progression or fibrosis-related diseases.^222–225^ The ECM contains various matrix proteins, especially fibrillar proteins such as FN and collagen, that have specific domains allowing them to interact with one another as well as cell receptors.

The ECM undergoes maturation and remodeling under physiological and pathogenic conditions. Alterations in the composition, structure, and conformation of matrix proteins are clinically relevant as they impact the overall tissue structure and cellular behavior. During wound repair, the activation of fibroblasts to myofibroblasts occurs at the highly tensed growth front which comprises stretched FN deposited by cells.^226^ As time passes, the more mature tissue on the interior comprises fibroblasts rather than myofibroblasts, where the abundance of collagen stabilizes FN fibers in a low-tension state. Therefore, fibroblast–myofibroblast activation is transient and regulated by cell-induced tension-matrix reciprocity.

Different conformational states of FN coexist.^227^ The structure of FN diffusively bound to cells is compact, while FN in matrix fibrils is highly extended. Although FN in cell-associated clusters before fibril formation has an extended structure, it is less extended than fibrillar FN. FN fibrillogenesis occurs *via* cell traction forces that induce the stretching and subsequent unfolding of specific modules in the FN structure (FNIII) *via* integrin receptors, exposing potential cryptic sites that promote FN self-association.^228–230^ During development, wound healing, and cancer progression, active FN fibrillogenesis is necessary and acts as a provisional template for collagen I deposition.^85,231^ Although, in the absence of FN, α_11_β_1_ and α_2_β_1_ integrins enhance the nucleation of collagen I, no well-organized collagen network is developed.^232^ Collagen I preferentially colocalizes with FN fibrils in a relaxed conformation rather than strained FN fibers because further FN stretching destroys the multivalent binding motif for collagen I.^85^ Once assembled, mature collagen fibers bear the overall tension and FN conformation relaxes from its previously strained state. As such, there is a conformational-dependent reciprocal interaction between FN and collagen I.

**Fig. 7:**
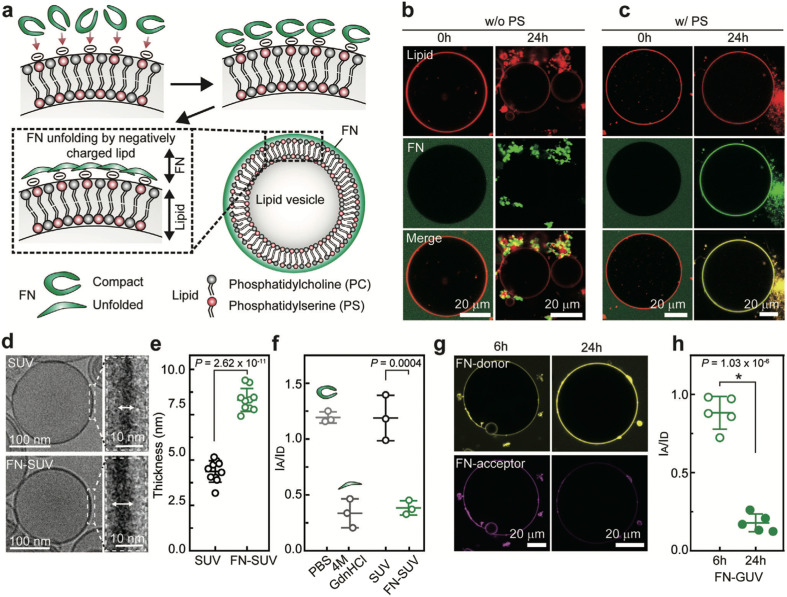
A schematic representation of conformational changes of FN upon adsorption on negatively charged vesicles. a) Demonstrating how negatively charged surfaces of vesicles induce binding and subsequent stretching of FN. b and c) Confocal images of GUV-FN binding; FN tends to form insoluble aggregates on the surface of neutral GUV (b); FN tends to attach evenly on the surfaces of negatively charged GUVs (c). d) Cryo-TEM images of an SUV and its membrane without FN (upper picture) and after incubation with FN (lower picture). e) Indicating a significant increase in the thickness of the SUV membrane after FN incubation. f) FRET data showing the intensity ratio of *I*_A_/*I*_D_ for double-labeled FN diluted in PBS, 4 m GdnHCl, or sucrose, or incubated with SUVs. *I*_A_ and *I*_D_ are the acceptor and the donor fluorescence intensity, respectively. g) Confocal imaging of single-vesicle FRET reveals the donor and acceptor intensities in GUVs after 6 and 24 hours of incubation with FN. h) Single-vesicle FRET demonstrating the intensity ratios *I*_A_/*I*_D_ after 6 and 24 hours of incubation with FN and GUVs. Reprinted with permission from ref. 233. Copyright 2021. John Wiley & Sons.

In this context, controlling and elucidating the structure and conformation of matrix proteins on NPs is likely to have significant implications for modulating cellular behavior and thereby providing insights into wound healing, cancer metastasis, and fibrotic-related disease. The negatively charged, small unilamellar lipid vesicles (SUVs, 200 nm) and analogous giant unilamellar vesicles (GUV, 20–30 μm) with the same composition selectively bind FN and unfolds it similar to that of FN treated with 4 M guanidine hydrochloride (Fig. 7).^233^ The stretched FN on the surface of GUVs has an anti-bacterial effect. The interaction of stretched FN on the surface of SUVs *via* integrin α_5_ receptors of human neonatal dermal fibroblast (HDFn) cells increases cell adhesion, migration, and ECM formation. FN-SUVs also reduce inflammation and swelling of the colon in rats with ulcerative colitis.

Reduced graphene oxide (RGO) with a more hydrophobic and less negatively charged surface at pH 7.4 adsorbs more FN than GO.^234^ The weakly adsorbed FN on the hydrophilic surface of GO undergoes conformational changes and possesses an elongated fibrillar structure, exposing its cryptic binding sites such as cysteine groups and RGD motif. In contrast, RGO adsorbs FN in a compact structure with buried bioactive sites. In the absence of serum, the elongated fibrillar structure of FN on GO increases stem cell attachment and spreading *via* interactions between the RGD binding site of FN and α_5_β_1_ and α_V_β_3_ integrins. Also, in the presence of serum, elongated fibrillar FN on GO adsorbed more serum proteins and growth factors, forming a rigid layer and ECM-rich-like microenvironment on GO, promoting stem cell differentiation. In contrast, compact FN on RGO weakly interacted with serum proteins, developing a flexible layer and decreasing stem cell differentiation. This is consistent with previous results highlighting the optimum conformation and orientation of FN on hydrophilic and negative substrates. Hydrophilic surfaces expose the active cell-binding sites of FN, resulting in higher integrin affinity and cell adhesion compared to hydrophobic surfaces that can strongly denature FN and disrupt its native structure.^235–237^ For instance, the adsorption of FN on hydrophilic glass promotes FN extension due to the competition of surface charges with intramolecular electrostatic interactions, leading to greater fibroblast cell adhesion and growth.^238^ However, despite disrupting the secondary structure of FN, hydrophobic fluoroalklysilane-derivatized glass (fluorosilane) adsorbed FN tightly in its compact overall shape, limiting its conformational flexibility around integrin-binding sequences and thereby reducing integrin-binding affinity. Therefore, controlling the extent to which NPs induce conformational changes in proteins should be carefully considered, as it could exert adverse effects on the biological activity of proteins.^239^ For instance, conformational changes of FN in the presence of nanodiamonds lead to denaturation, multimerization, and aggregation of FN, which could cause FN to lose its functionality *in vivo*.

Anatase TiO_2_ and rutile TiO_2_ films adsorb a similar amount of FN due to their similar hydrophilicity.^240^ However, FN adsorption on anatase TiO_2_ films, which have more hydroxyl groups than rutile TiO_2_ films, leads to better adhesion, proliferation, differentiation, mineralization and osteogenesis-related gene expression. This is due to the greater amount of hydroxyl groups on anatase TiO_2_ films inducing FN unfolding to a favorable conformation for exposing its RGD binding site. Similarly, the adsorption of FN and collagen I on poly(sodium styrenesulfonate) (poly(NaSS)) *via* hydrophilic and electrostatic interactions results in the highest cell adhesion.^241^ The conformational and orientational changes of FN on the surface of Poly(NaSS) cause FN to expose its active binding sites, namely, both RGD and the HB domains, for cell attachment. This leads to higher integrin-mediated MC3T3-E1 cell adhesion. FN adsorbs in a higher density on nanowire structures added to titanium (Ti) plasma-sprayed Ti implants (NW-Ti), which have higher hydrophilicity and specific surface area, as well as less negative zeta potential, compared to nanonest-Ti (NN-Ti), nanoflake-Ti (NF-Ti), and Ti controls.^242^ The higher FN adsorption on NW-Ti, despite its hydrophilicity, can be explained by the higher specific area of NW-Ti, which plays a more critical role in FN adsorption than its hydrophilicity. FN adsorption and subsequent conformational changes on NW-Ti promote the accessibility of the RGD binding site of FN, increasing stronger adhesion, spreading, and osteogenic differentiation of bone marrow-derived mesenchymal stem cells (BMSCs) *via* the interaction between the RGD binding site of FN and α_5_β_1_ integrins.

The conformational state of matrix proteins is also relevant to matrix stiffening, which is implicated in the progression of cancer. For instance, exposing adipose stromal cells to breast cancer-cell secreted factors leads to the deposition of a high amount of stiff and unfolded FN.^222^ The conformational changes of tumor-associated FN increase the spatial separation between the integrin-binding sequence of FN (FNIII_9_–FNIII_10_), forcing cells to switch from α_5_β_1_ to α_v_β_3_ binding, which is insensitive to the increased distance between FNIII_9_ and FNIII_10_. The integrin switch in cells followed by an increase in the release of proangiogenic factor (VEGF) likely promotes vascularization and breast cancer tumor growth. MMPs have a major role in remodeling tumor stroma by degrading and remodeling FN, exposing its collagen binding sites, which increases collagen I fibrillogenesis.^231^ Mature, dysregulated collagen I fibers in the tumor stroma partially stabilize FN against cell exerted unfolding, which, in turn, increases collagen I deposition. Therefore, the initially stretched, unfolded FN matrix in tumor stroma remodels into a matrix rich in thick collagen I. The more relaxed structure of FN, stabilized by the presence of collagen I, exposes the Hep2 domain of FN, favoring the binding of VEGF. Thus, the conformational changes of FN in tumor stroma during ECM remodeling impact the secretion of VEGF as well as its sequestration over time, modifying proangiogenic signaling.

GO and porous GO (pGO) films selectively promote the proliferation and spread of metastatic (MDA-MB-231) and nonmetastatic (MCF-7) breast cancer cells, while having no effect on noncancer breast epithelial cells (MCF-10A).^243^ In contrast, graphene films have the highest cytotoxicity and do not have accelerated proliferation effects on cells. This is attributed to the higher adsorption of FN and insulin on GO and pGO than on graphene films. The adsorbed insulin maintains its conformational stability regardless of the type of graphene material used. In contrast, adsorbed FN on GO and pGO undergoes secondary structure conformational changes from random coil to β sheet due to oxygenated groups on GO and pGO films mediating strong electrostatic interactions between FN and hydrophilic GO materials, exposing FN integrin-binding sites for interaction with cancer cells.

### b. Leveraging protein corona conformation to control immune responses

The PC confers NPs a new immunological identity that directly impacts their blood circulation time and macrophage evasion.^145^ The presence of opsonins, such as complement factors, immunoglobulins, acute phase proteins, and coagulation factors, in the PC, has been correlated to the clearance of NPs from blood circulation *via* the opsonin-cognate receptors expressed on the phagocytic surface.^143,145,244–247^ In contrast, the presence of HSA and apolipoproteins in the PC provides NPs with a stealth effect, promoting prolonged circulation time.^248–250^ PCs can either upregulate or downregulate the release of cytokines depending on the biological environment where the PC is formed, the properties of NPs, and the immune cell line.^251^ Therefore, the PC can be leveraged to either suppress or induce the immune system, where the latter is used for cancer immunotherapy to clear cancer cells.^19,251,252^ For instance, the formation of an intracellular PC consisting of a signal transducer and activator of transcription 3 (STAT3) on the surface of GO nanosheets in the cytoplasm of tumor-associated macrophages (TAM) prevents the immunosuppressive phenotype of TAM.^253^ The interaction between STAT3 and GO nanosheets inhibits STAT3 activation by preventing its entrance to the nucleus, triggering M1 macrophage polarization, and facilitating tumor immunotherapy.

Interestingly, although the PC of SiO_2_ NPs incubated in human plasma is enriched with immunoglobulins, complement factors, and coagulation proteins, PC-SiO_2_ NPs are protected against uptake by RAW 264.7 macrophage.^248^ This could be due to the organization of proteins in the PC, where dysopsonin proteins such as HSA and lipoproteins block the binding sites of opsonin proteins by trapping them in the inner layer of the PC. Or this could be attributed to the unfolding of opsonin proteins on the surface of SiO_2_ NPs in a way that their epitopes are buried inside. This suggests that besides determining the composition of the PC, the organization and conformation of proteins associated with the PC should be considered for predicting or controlling the fate of NPs *in vivo*.

**Fig. 8:**
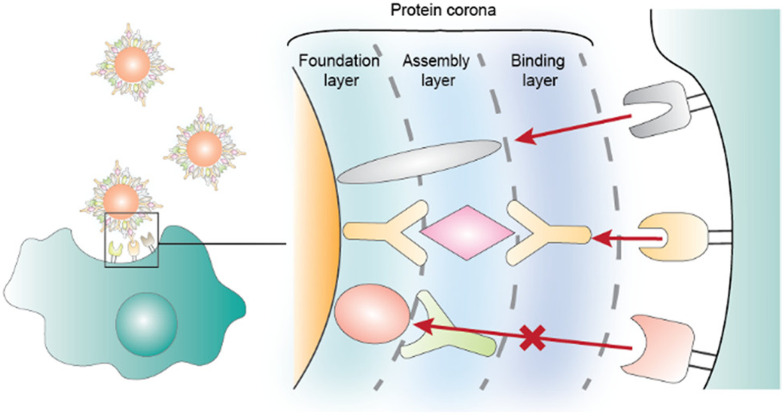
Hypothesized mechanism for the structural-dependent function of the PC, where the PC contains three layers, including the foundation layer, proteins adsorbed on the surface of NPs, the assembly layer, proteins with no binding functionality being sterically covered by other proteins, and the binding layer, responsible for binding to cell receptors. Reprinted with permission from ref. 254. Copyright 2020. American Chemical Society.

A recent study shows that manipulating the organization of proteins in the PC controls their binding functionality, thereby changing NP–macrophage cell interactions.^254^ The binding property of IgG in the PC is altered using a workflow termed knock-out assisted binding activity modification (KABAM). It is well known that IgG serves as a bridge between PC and C3 opsonization.^255^ The number of binding functional IgG increases with the depletion of C3/C4, leading to an increase in the binding of NPs treated with C3/C4 depleted serum to macrophages, implying that C3 and C4 non-specifically block the binding function of IgG.^254^ Conversely, the binding property of IgG decreases as its binding partner, antithrombin III (ATIII), is depleted from the PC. Subsequently, NPs treated with an ATIII depleted PC shows less binding to macrophage cells, suggesting that in a full serum, ATIII acts as a supporting layer for IgG. These results propose that adsorbed proteins in the PC assemble in multiple layers containing protein–protein interactions, and the outermost layer has a significant role in NP–macrophage cell interactions (Fig. 8).

Random copolymer brushes with coating composition consisting of 75% hydrophilic sulfobetaine methacrylate (SBMA) and 25% amphiphilic oligoethylene glycol methacrylate (OEGMA) can stabilize the folded structure of FNIII_8–10_, which is a relevant model for full-length FN.^256^ Stabilized FNIII_8–10_ adsorbed on 75% SBMA/25% OEGMA diminishes the release of TNF-α and IL-6 by RAW 264.7 macrophages. This implies that tuning the heterogeneity of biomaterials allows for control over the dynamic and transient structure of FN, thereby preventing macrophage activation. However, the mechanism by which mixed brushes stabilize the native conformation of FNIII_8–10_ has yet to be determined. The interaction of HSA, Tf, and IgG with two-dimensional (2D) nanosheets (NSs) changes the secondary structure of proteins by decreasing their α-helix content and increasing their β-sheets, except for IgG, which has the highest β-sheet content and exhibits the least significant secondary structural changes on the surface of NSs.^249^ Despite the minimal secondary structural changes of IgG on NSs, IgG-coated NSs have the highest cellular uptake into macrophages and trigger a stronger inflammatory response by promoting more release of cytokines such as TNF-α and IL-6 than other protein–NS complexes. Furthermore, FcγRs receptors, highly expressed on the cytoplasmic membrane of macrophages, are responsible for the detection and uptake of IgG-coated NSs as well as the activation of the NF-κB pathway, which is critical in regulating inflammatory effects. Tf and HSA-coated NSs with reduced uptake into macrophages can be used for hiding NPs from macrophages. In contrast, IgG and FBG-coated NPs that cause the most inflammatory reactions can be employed for immunotherapy purposes.

Fullerol NPs neither cause notable structural changes in IgG nor change the secretion of TNF-α in THP-1 cells exposed to IgG-fullerol.^257^ In contrast, Lyz binding to the same NPs increases Lyz β-sheet formation as well as amyloid fibrillation and greatly elevates cytokine production in THP-1 cells. The attachment of IgG and alpha1 acid-glycoprotein to PEGylated carbon nanotubes (CNTs) significantly changes their secondary structure, where the extent of the secondary structural changes increases with decreasing diameter of the CNTs.^258^ This increases reactive oxygen species (ROS) levels and triggers the release of proinflammatory cytokines in J774A.1 macrophages. On the contrary, regardless of the size of CNTs, conjugated FBG and vitronectin neither undergo significant conformational changes nor trigger immune responses. When injected into BALB/c mice, the unfolded proteins on the nanotubes activate the innate and adaptive immune systems, especially in the spleen. The altered structure of unfolded proteins in the PC of NPs can be applied to an immune activation approach for cancer immunotherapy, in which unfolded proteins can boost immune activity in the tumor target tissue and, thus, eliminate tumor cells.

While conformational changes of a protein on a substrate with a specific chemical property trigger the secretion of pro-inflammatory cytokines, the conformational changes of the same protein on other surfaces may result in no immune response, suggesting the significance of surface property as well as the type of conformational change of proteins. The complexity of immune responses has also been displayed in another study, where the pre-adsorption of HSA on four substrates with varying surface chemistries suppresses the release of pro-inflammatory responses while inducing the secretion of anti-inflammatory cytokines.^259^ The interaction of immune cells with HSA *via* scavenger receptors occurs through binding to peptide sequences of HSA, which are hidden unless its native structure unfolds. The plasma polymerized allylamine (AA) and plasma deposited 2-methyl-2-oxazoline (pOX) surfaces induce significant secondary structural changes in HSA. However, AA surfaces pre-coated with HSA have higher dTHP-1 macrophage cell adhesion than POX pre-coated with HSA. This suggests that macrophage adhesion depends on not only the level of secondary structural loss of adsorbed protein but also the type of conformational change. While the pOX substrate induces the most secondary structural loss and unfolding in HSA, this surface leads to the least secretion of pro-inflammatory cytokines, implying that there is not always a correlation between the expression of immunological markers, the amount of adsorbed HSA, and the level of surface-induced unfolding.

Macrophages are part of the innate immune system, and depending on their specific phenotype, they have different roles in the wound-healing process, such as the phagocytosis of local debris and pathogens, the regulation of the entire inflammation process, and support of cell proliferation and tissue restoration following injury.^41,260^ Despite the beneficial impact of macrophages on the healing process, macrophages have a role in chronic wounds by retaining their M1 phenotype, thereby maintaining pro-inflammatory characteristics.

The adsorption of the dynamic PC on MNP-infiltrated hydroxyapatite (MHAp) scaffolds correlates with immune-modulated bone wound healing.^261^ MHAp scaffolds with small magnetic fields adsorb inflammatory and immune-related proteins and suppress chronic inflammatory responses while significantly promoting acute inflammatory responses. This results in the recruitment of immune cells secreting cytokines and factors crucial for extracellular remodeling and bone wound healing. Controlling the conformation of proteins associated with the PC on the surface of NPs and biomaterials can be exploited as a strategy to induce immune responses or *vice versa*. When in the biological fluid or interstitial environment, NP-induced conformational changes of proteins associated with the PC may expose hidden protein epitopes and trigger an immune response.^262^ For example, the binding of FBG to 5 nm, negatively charged poly(acrylic acid)-conjugated AuNPs results in the unfolding of FBG, exposing the C-terminus of its γ chain, which interacts with the Mac-1 integrin receptor of THP-1 cells.^263^ This enhances the NF-kB signaling pathway, resulting in the release of inflammatory cytokines. However, such an effect is not observed for NPs larger than 20 nm, which adsorb a greater amount of FBG because steric hindrance prevents FBG from unfolding and exposing the C-terminus of its γ chain. FBG binds to both positively and negatively charged AuNPs with a high affinity.^264^ However, although the binding of FBG to negatively charged AuNPs triggers the release of TNF-a and IL-8 from the B-cell derived THP-1 cells, FBG binding to positively charged NPs does not affect cytokine release, suggesting a different orientation of FBG on alternately charged NPs. Separately, the surface of the NW-Ti prevents FBG from unfolding, preserving its native secondary structure, which is inactive to immune cells because sequences interacting with the Mac-1 integrin receptor are hidden in the native structure of FBG.^242^ As a result, M2 macrophages accumulate on the surface of NW-Ti, enhancing the immunosuppressive function of BMSCs.

## 5. Conclusion and outlook

To bridge the gap between experimental studies and clinical translation of nanomedicines, a growing body of studies has emerged to investigate the role of the PC as it affects the fate and biological functionality of NPs. While most studies focus on the composition of the PC and its correlation with the biological activity of NPs, more in-depth details are needed to fully understand the complex interrelationship of NPs and PCs. NPs can induce conformational changes in adsorbed proteins, which have consequences for the functionality of proteins and the fate of NPs. Additionally, NP-induced conformational changes of a PC can cause undesired effects, such as inducing an unwanted immune response, impairing enzyme activity, and decreasing the colloidal stability of the NPs. At the same time, this can be exploited for therapeutic purposes, which could be a new era for preventing cancer progression or improving tissue regeneration.

In this review, we have introduced biological factors that alter the conformation of proteins on the surface of NPs, potentially mitigating undesired biological responses while enhancing the efficacy of the nanomedicine. Then, we reviewed the impact of four types of NPs frequently used for biomedical applications on the conformational changes of proteins in the PC. We also discuss the extent to which controlling the conformation of proteins in assembled matrices in tissues is essential for preventing cancer progression or improving tissue regeneration, as well as the impact of protein conformation on immune responses.

Most studies focus on evaluating the hard PC while paying less attention to the soft PC. This is likely due to a lack of technology and techniques for preserving and separating NPs with the loosely bound soft PC. Although most proteins associated with the soft PC maintain their native conformation because they interact with hard PC *via* protein–protein interactions rather than directly accessing the surface of NPs, a few can still access the surface of NPs due to protein exchange, thereby undergoing structural changes.^86,219^ The conformational changes of proteins associated with the soft PC likely have biological relevance for the fate and functionality of NPs. As such, the soft PC remains an unexplored yet essential domain.

Although most *in vitro* studies have focused on tuning the surface property of NPs for controlling NP–PC interaction and enhancing the biological functionality, future PC-related research should study the conformational changes of proteins under more relevant biological conditions by considering all relevant biological factors influencing PC *in vivo*. For instance, often overlooked is the impact of flow for intravenously administered NPs, which affects the composition and structure of the PC. In this context, the exposure order of proteins to NPs is also relevant because NPs experience different organs and fluids depending on their route of administration. Predicting or managing the conformational changes of proteins associated with PC is further complicated by the possibility of interconnection between biological factors and material properties. Recently, there have been efforts to evaluate *in vivo* PC formed in human systemic circulation by recovering the PC–NPs from the blood circulation of patients.^265^ Within the context of leveraging PC for therapeutic purposes, material properties can be tuned to control the conformation and orientation of adsorbed proteins, exposing or hiding specific binding sites in order to modulate immune responses. Such a strategy can be used for prolonging the blood circulation time of NPs by evading macrophages, cancer immunotherapy, or even immune-modulated wound-healing.

Efforts to control the conformational state and orientation of proteins by tuning the material properties and involved biological factors would considerably enhance NPs' therapeutic impact.

## Abbreviation

PC: Protein corona
NP: Nanoparticle
FBG: Fibrinogen
FN: Fibronectin
ECM: Extracellular matrix
pI: Isoelectric point
FRET: Fluorescence resonance energy transfer
AuNPs: Gold nanoparticles
MNPs: Magnetic iron oxide nanoparticles
SLNs: Solid lipid nanoparticles
Hb: Hemoglobin
Lyz: Lysozyme
β-LG: β-Lactoglobulin
oxyHb: Oxyhemoglobin
Tf: Transferrin
ApoTf: Iron-free Tf
β2m: β2-microglobulin
Cit-AuNPs: Citrate-stabilized AuNPs
GO: Graphene oxide
MD: Molecular dynamics
HSP90: Heat shock protein 90
gly: Glycine
lys: Lysine
glu: Glutamic acid
ser: Serine
AuNRs: Gold nanorods
ApoA1: Apolipoprotein A1
HRG: Histidine-rich glycoprotein
LC: Low-serum corona
HC: High-serum corona
HAp: Hydroxyapatite
αS: Alpha-synuclein
MMPs: Matrix metalloproteinases
NSCLC: Non-small cell lung cancer
PEG: Polyethylene glycol
A2M: Alpha-2-macroglobulin
vLDL: Very-low-density lipoprotein
HDL: High-density lipoprotein
ngTf: Non-glycosylated recombinant form
GSH-AgNPs: Glutathione silver NPs
NC: Nanocluster
cyt c: Cytochrome complex
DHLA: Dihydrolipoic acid
GSH: Glutathione
ChT: α-Chymotrypsin
FA: Folic acid
BCA: Bovine carbonic anhydrase
NR: Nanorod
NS: Nanostar
NSP: Nanospheres
FXII: Coagulation factor XII
IDPs: Intrinsically disordered proteins
BHb: Bovine Hb
vWF: von Willebrand factor
IONPs: Iron oxide nanoparticles
SPIONs: Superparamagnetic iron oxide nanoparticles
MIONPs: Magnetic iron oxide NPs
HM: Hematite
RA: Rosmarinic acid
Arg: Arginine
β-LG: Beta-lactoglobulin
STP: Sodium triphosphate
TSC: Trisodium citrate
TLC: Trilithium citrate
CPC: Cetyl pyridinium chloride
QDs: Quantum dots
CdTe: Cadmium telluride
TGA: Thioglycolic acid
MPA: Mercaptopropionic acid
MoS_2_: Molybdenum disulfide
CDs: Carbon nanodots
PEI: Polyethyleneimine
RGO: Reduced graphene oxide
Ti: Titanium
VEGF: Vascular-endothelial growth factor
TAM: Tumor-associated macrophages
SBMA: Sulfobetaine methacrylate
OEGMA: Oligoethylene glycol methacrylate
NSs: Nanosheets
CNTs: Carbon nanotubes
ROS: Reactive oxygen species
AA: Allylamine
pOX: Plasma deposited 2-methyl-2-oxazoline

## Author contributions

G. B. and K. W. conceptualized this review. G. B. and M. S. P. drafted this review. M. S. P, K. L. S., and S. J. S. created the figures. K. W. and M. J. M. revised this review. All the authors reviewed the results and approved the final version of the manuscript.

## Conflicts of interest

There are no conflicts to declare.

## Supplementary Material
